# A Microbiome-Based Index for Assessing Skin Health and Treatment Effects for Atopic Dermatitis in Children

**DOI:** 10.1128/mSystems.00293-19

**Published:** 2019-08-20

**Authors:** Zheng Sun, Shi Huang, Pengfei Zhu, Feng Yue, Helen Zhao, Ming Yang, Yueqing Niu, Gongchao Jing, Xiaoquan Su, Huiying Li, Chris Callewaert, Rob Knight, Jiquan Liu, Ed Smith, Karl Wei, Jian Xu

**Affiliations:** a Single-Cell Center and Shandong Key Laboratory of Energy Genetics, Qingdao Institute of BioEnergy and Bioprocess Technology, Chinese Academy of Sciences, Qingdao, Shandong, China; b Procter & Gamble Beijing Innovation Center, Beijing, China; c Procter & Gamble Singapore Innovation Center, Singapore, Singapore; d Office of General Affairs, Chinese Academy of Sciences, Beijing, China; e Department of Molecular and Medical Pharmacology, University of California at Los Angeles, Los Angeles, California, USA; f Center for Microbiome Innovation and Departments of Pediatrics, University of California at San Diego, La Jolla, California, USA; g Center for Microbial Ecology and Technology, Ghent University, Ghent, Belgium; h Procter & Gamble Mason Business Center, Mason, Ohio, USA; i University of Chinese Academy of Sciences, Beijing, China; Pacific Northwest National Laboratory

**Keywords:** atopic dermatitis, personalized skin care, skin microbiome, spatial variation, suboptimal health

## Abstract

A quantitative and objective indicator for skin health via the microbiome is of great interest for personalized skin care, but differences among skin sites and across human populations can make this goal challenging. A three-city (two Chinese and one American) comparison of skin microbiota from atopic dermatitis (AD) and healthy pediatric cohorts revealed that, although city has the greatest effect size (the skin microbiome can predict the originated city with near 100% accuracy), a microbial index of skin health (MiSH) based on 25 bacterial genera can diagnose AD with 83 to ∼95% accuracy within each city and 86.4% accuracy across cities (area under the concentration-time curve [AUC], 0.90). Moreover, nonlesional skin sites across the bodies of AD-active children (which include shank, arm, popliteal fossa, elbow, antecubital fossa, knee, neck, and axilla) harbor a distinct but lesional state-like microbiome that features relative enrichment of Staphylococcus aureus over healthy individuals, confirming the extension of microbiome dysbiosis across body surface in AD patients. Intriguingly, pretreatment MiSH classifies children with identical AD clinical symptoms into two host types with distinct microbial diversity and treatment effects of corticosteroid therapy. These findings suggest that MiSH has the potential to diagnose AD, assess risk-prone state of skin, and predict treatment response in children across human populations.

**IMPORTANCE** MiSH, which is based on the skin microbiome, can quantitatively assess pediatric skin health across cohorts from distinct countries over large geographic distances. Moreover, the index can identify a risk-prone skin state and compare treatment effect in children, suggesting applications in diagnosis and patient stratification.

## Abstract (precis)

MiSH, which is based on the skin microbiome, can quantitatively assess pediatric skin health across cohorts from distinct countries over large geographic distances. Moreover, the index can identify a risk-prone skin state and compare treatment effect in children, suggesting applications in diagnosis and patient stratification.

## INTRODUCTION

A central goal of human microbiome projects is to diagnose and predict host states via the microbiome (1, 2). The skin, our largest organ and a first line of environmental exposure, hosts a microbiome that is site specific, host specific, and environment specific (3, 4). Particular skin symbionts modulate the host immune response, physiology, and development (5–8). Therefore, the prospect of exploiting the skin microbiome for health protection, disease treatment, or personal care has attracted great interest (9). The skin microbiome is known to differ between human populations (10, 11); therefore, whether the skin microbiome can serve as an indicator of skin health that applies across large geographic ranges remains largely unknown (12, 13).

This challenge can be traced to the characteristics of human skin microbiota. The dominant types of resident skin bacteria appear relatively stable, and less abundant types of bacteria account for most of the variability (4). Within an individual, composition of the skin microbiota is determined primarily by body site. Within a skin zone, temporal variability in an individual is small compared to interpersonal variability (3, 4, 14, 15). However, how this pattern of spatial variation manifests itself remains poorly understood, particularly in the context of perturbation by disease or medications. Moreover, changes in pathogenic microbiota across individuals, cities, and even at the global scale is largely unknown (4, 16, 17). Furthermore, for many microbiome-wide association studies, notably those from the gut, applying models of microbial disease biomarkers trained in one population to other populations has typically been unsuccessful (18), which greatly limits the potential of the microbiome for diagnosis and treatment-oriented patient stratification.

Atopic dermatitis (AD), a chronic and relapsing inflammatory skin disorder associated with skin barrier impairment, affects 15 to 30% of children (5% of the general population) worldwide and has been rapidly increasing in prevalence, especially with children (19, 20). AD is a heterogeneous disease of different subtypes and with varied and sometimes evasive clinical manifestations (21). Disease severity is typically diagnosed by physicians via visual observation and diagnosis of disease signs, including color change, pruritus, and swollen and cracked skin, generally done with the well-validated Scoring Atopic Dermatitis Index (SCORAD) (22). Other diagnosis measures include the Eczema Area and the Severity Index (EASI) score (23) or the objective SCORAD (24). In addition, several serum biomarker assays, such as thymus and activation-regulated chemokine (TARC) assay (CC chemokine 17 [CCL17]), pulmonary and activation-regulated chemokine (PARC) assay (CCL18) (25–28), Staphylococcus aureus enterotoxin assay (29), etc. are available, yet they are typically invasive. On the other hand, the AD state has been associated with change in the skin microbiome, e.g., the presence or enrichment of S. aureus (4, 15, 30). These microbiome-based findings are enabling new opportunities for better assessment or prediction of the disease state, which can potentially overcome the shortcoming of traditional clinical scores or supplement them, particularly in allowing comparison among patients, examiners, or studies, and in objective design and administration of skin therapy and care regimens (31).

On the other hand, it remains elusive whether and how skin microbiome plays a role in AD treatments (30), which aims to reduce symptoms (pruritus and dermatitis), prevent exacerbations, and minimize therapeutic risks. Standard treatment modalities for AD have centered around the use of topical anti-inflammatory preparations and moisturization of the skin (e.g., corticosteroids, calcineurin inhibitors, and crisaborole), and patients with severe AD may require phototherapy or systemic treatment (for instance, oral cyclosporine [32, 33]). Topical calcineurin inhibitors are potentially linked to cancer (34), crisaborole remains uncertain in efficacy (35, 36), and cyclosporine may induce side effects, including nephrotoxicity, hypertension, hypertrichosis, etc. (37). Therefore, corticosteroids can serve as a starting point for probing role of the skin microbiome in AD treatment, since they are recommended as first-line treatment for AD (38).

In this study, we compared skin microbiota across the body (mainly from the forearm and shank, with the remaining from seven additional skin sites; see Table S1 in the supplemental material) from healthy and AD active children (3 to 12 years old) from three cities (Beijing and Qingdao from China and Denver from United States), and tracked their subsequent response to skin care treatment. We showed that although city has the greatest effect size, a Microbial Index of Skin Health (MiSH) is generally applicable to populations across large geographical distances. Moreover, in AD, we confirmed that the microbiome dysbiosis is extended across whole body surface, as nonlesional skin sites of the patient harbor a distinct but lesional state-like microbiome. Intriguingly, pretreatment MiSH classifies children in the Beijing cohort with identical AD clinical symptoms into two host types with distinct disease severity and sensitivity to corticosteroid therapy (in which corticosteroid-containing ointment was applied on skin surface). Although their reproducibility and generalizability need to be demonstrated in larger cohorts and various populations, our results indicate that, via MiSH, the skin microbiome may potentially serve as a generally applicable, quantitative proxy to diagnose AD, be able to compare the efficacies of AD care products, and be able to predict AD treatment response in children.

10.1128/mSystems.00293-19.8 TABLE S1 Effect size of host ID, body site, biological sex, age, city, and disease status on the variation of skin microbiome. Based on the inclusion criteria, both mild and moderate AD were included in each of the cohorts. Download Table S1, DOCX file, 0.05 MB. Copyright © 2019 Sun et al. 2019 Sun et al. This content is distributed under the terms of the Creative Commons Attribution 4.0 International license.

## RESULTS

### Experimental design that compared healthy and AD skin microbiota at various spatial scales.

**FIG 1:**
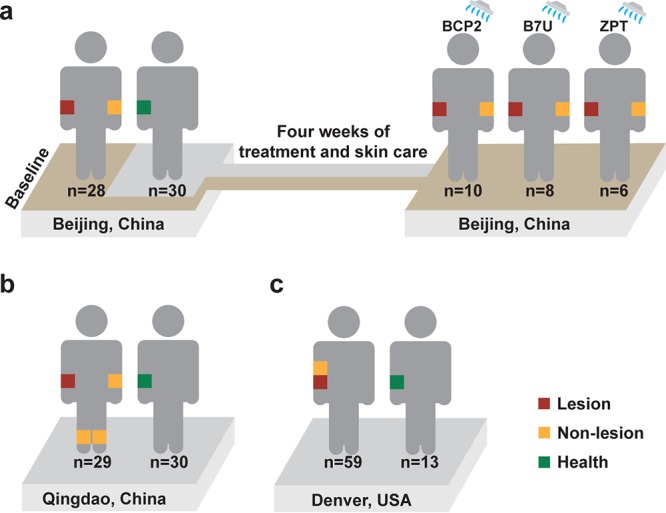
(a to c) Experimental design that sampled skin microbiota from AD-active children and healthy controls in the two Chinese cities of Beijing (a) and Qingdao (b) and the American city of Denver (c). In diseased children, skin microbiota from both lesional sites and the nonlesional sites were collected (Fig. 5a for details). In healthy children, the sampling sites were matched with the lesional sites of patients. For the Beijing cohort, skin microbiota before and after various treatment regimens was sampled.

To test whether healthy and AD-active skin microbiota are distinct over wide geographical areas, we established two cohorts. The first cohort consists of 28 children age 4 to 12 years from the Chinese city of Beijing (“Beijing cohort”) who were suffering from mild to moderate AD (Fig. 1a) (Materials and Methods). A second cohort of age-matched children was recruited from Qingdao, a coastal Chinese city at the West Pacific rim 650 kilometers southeast of Beijing. The “Qingdao cohort” consists of 29 pediatric patients suffering from mild to moderate AD and 30 healthy subjects (Fig. 1b) (Materials and Methods), who were screened using selection criteria identical to those for the Beijing cohort. Furthermore, we compared the results to a third cohort that consists of 59 AD-active and 13 healthy children (4 to 12 years old) from the Denver, CO (“Denver cohort”) (Fig. 1c) (Materials and Methods).

For the Beijing and Qingdao cohorts, our study design also compared lesional and nonlesional skin sites at multiple locations across the body surface (Table S1). Moreover, microbiome changes were compared between patients at their first visit and at 4 weeks later (i.e., after the treatment). Specifically, for the Beijing cohort that used corticosteroids as treatment, clinical symptoms were greatly reduced (ΔSCORAD = 25.7 ± 7.5) in 16 patients and partially relieved (ΔSCORAD = 6.5 ± 6.7) in the remaining 14 patients. Their skin microbiomes at both lesional and nonlesional sites were sampled at baseline and posttreatment (Fig. 1a) (Materials and Methods).

### City of origin affects skin microbiome more than the AD status.

The skin microbiome is affected by location on the human body, disease status, and host individuality (39). Thus, we assessed the effect size of AD at the four geographical scales of skin site, host individual, city, and continent, via the Beijing (China), Qingdao (China) and Denver (USA) cohorts. Age and biological sex were similar among the three cohorts (*P = *0.086, Kruskal-Wallis test). Within each of our city-specific cohorts, AD status had a larger effect size than did skin site, host identification (ID), age, or sex (Table 1). However, analyses over the three cities revealed that geographic location and AD status both greatly affected the skin microbiota, despite a larger effect size of the former (F = 25.93 versus 18.72; Table 1).

**TABLE 1:** Details of participants and samples from Beijing, Qingdao, and Denver

Factor	F value (Adonis)	*P* value
City	25.93	0.001
Status^*a*^	18.72	0.001
SCORAD	7.67	0.002
Site	1.82	0.036
Age	1.45	0.001
Individual	1.36	0.031
Biological sex	0.43	0.678

a Status, health status based on AD diagnosis.

**FIG 2:**
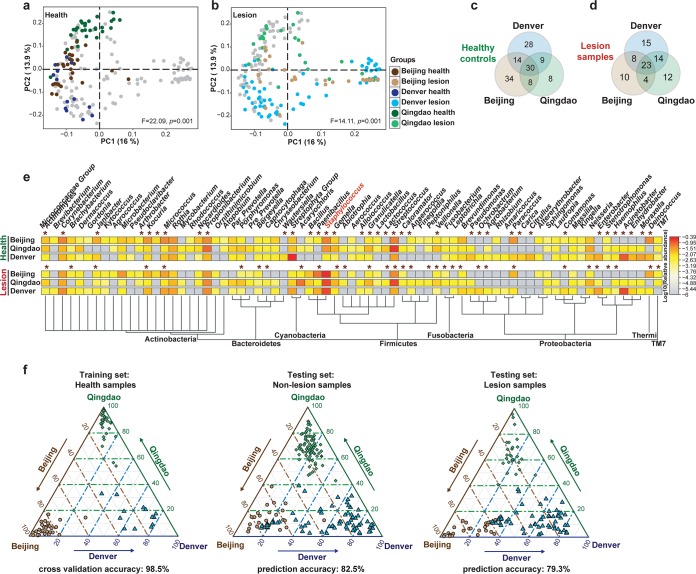
Diversity of healthy or diseased skin microbiota among the three cities. (a) PCoA of the healthy controls from the three cities. Within a healthy population, geographic (city) variation in skin microbiota is remarkable (F = 22.09, *P = *0.001). (b) PCoA of the lesional samples from the three cities. The between-city difference was reduced after AD infection (from F = 22.09 to F = 14.11), indicating that AD drives the convergence of skin microbiota from distinct cities. (c and d) Shared and specific bacterial genera among the cities, for healthy (c) and lesional (d) samples. (e) Heatmap of bacterial genera in both healthy and lesional skin (only those with relative abundance >1% were shown; *, significantly changed). (f) Skin microbiota predict city origin. Healthy samples were used as training set, with healthy (left), nonlesional (middle), and lesional (right) samples, respectively, as the testing set. In the ternary plot, the closer one sample is to an apex, the more likely it is predicted to be from that city. The likelihood to correctly predict Denver samples is lower than the other cities, and moreover, the Denver samples are more difficult to separate from Beijing than from Qingdao (f, middle diagram). The reason for this observation, however, is not clear.

To identify the bacteria contributing to city-specific signatures, we analyzed healthy and diseased skin microbiomes within each city and then compared the results. For healthy individuals, significant differences (β diversity) were found among Beijing, Qingdao, and Denver as shown via principal-coordinate analysis (PCoA) (Fig. 2a; F = 22.09, *P < *0.001, Adonis). The AD microbiota (i.e., the lesional samples from AD patients) also varied among cities (Fig. 2b, the same PCoA plot as Fig. 2a yet with a distinct color scheme; F = 14.11, *P < *0.001, Adonis), although the AD microbiota communities were more similar to each other than the non-AD microbiota at the city level (Fig. 2a and b). Moreover, the PC1 of PCoA was negatively correlated with Propionibacterium, Caloramator, Rothia, Prevotella, Nocardioides, Actinomyces, and Corynebacterium (rho = −0.62, −0.54, −0.53, −0.53, −0.52, −0.52, and −0.52, respectively; Fig. S1a), and positively correlated with Staphylococcus (rho = 0.54; Fig. S1a, red) and SCORAD (rho = 0.47; Fig. S1b). In addition, the PC1 appeared to be indicative of the disease status, as PC1 values were different between healthy and lesion samples (but not those between biological sex or among the three cities; Fig. S1c).

10.1128/mSystems.00293-19.1 FIG S1 The major driving force behind the PCoA of healthy and lesion samples from the three cities. (a) Correlation (via Spearman coefficient) between the various bacterial genera and PC1 in the PCoA. All those with absolute value of rho greater than 0.5 are shown. (b) Correlation between PC1 and SCORAD. (c) PC1 values of the samples are differentially distributed between healthy and lesion samples but are not associated with either city origin or biological sex. Download FIG S1, TIF file, 0.5 MB. Copyright © 2019 Sun et al. 2019 Sun et al. This content is distributed under the terms of the Creative Commons Attribution 4.0 International license.

In Beijing, Qingdao, and Denver, 90, 67, and 87 bacterial genera were identified, respectively (see Materials and Methods). For healthy samples, 30 genera were found in all of the cities, representing 63.2%, 73.4%, and 54.0% abundance, respectively, while for AD samples, 23 genera are shared, representing 83.3%, 71.2%, and 60.5% abundance, respectively (Fig. 2c and d). Among the healthy samples, 34 (Beijing), 8 (Qingdao), or 28 (Denver) were city-specific genera, and 38 shared genera (totally 60.1% in relative abundance, on average) have changed in relative abundance among cities (*P < *0.01, ANCOM (40) (Fig. 2e and Table S2); among lesional samples, 10 (Beijing), 12 (Qingdao), or 15 (Denver) city-specific genera were detected, with 41 (totally 63.1% in relative abundance, on average) altered in relative abundance among cities (*P < *0.01, Wilcoxon test) (Fig. 2e and Table S2).

10.1128/mSystems.00293-19.9 TABLE S2 Differentially distributed genera among the three cities of Beijing, Qingdao, and Denver in healthy and AD-active children. Download Table S2, DOCX file, 0.02 MB. Copyright © 2019 Sun et al. 2019 Sun et al. This content is distributed under the terms of the Creative Commons Attribution 4.0 International license.

City-specific bacterial markers from healthy samples overlapped by >50% those from AD samples (Fig. 2e; notably, *Staphylococcus* spp. vary among cities in healthy samples yet were of identical, enriched abundance in diseased samples, likely resulting from selection by disease). To test whether these city-specific markers can predict the city origin, we built classification models using random forests (RF) with the healthy samples as training set (Materials and Methods) (Fig. S2a). The city origin was predicted from healthy samples with 98.6% accuracy (Fig. 2f; left) by 10-fold cross validation. Moreover, models trained using healthy samples predicted the origin of both nonlesional and lesional samples with 82.5% and 79.3% accuracy, respectively (Fig. 2f; middle and right). Thus, city-specific differences in skin microbiome were consistent, irrespective of the health status.

10.1128/mSystems.00293-19.2 FIG S2 Selection of bacterial markers for the RF models. (a) Selection of bacterial markers for the models that predict city origin based on skin microbiota. The 20 genera with the most discriminating power are shown as a bar plot, which were selected as markers in the city classification model. The bar length at each row indicates the relative contribution of a genus to the RF model. Insets, relationship between the number of variables (i.e., genera) in the RF model and model performance. (b to d) Selection of bacterial markers for the AD diagnosis models in Beijing (b), Qingdao (c), or Denver (d). The bar length at each row indicates the relative contribution of a genus to the RF model. Insets, relationship between the number of variables (i.e., genera) in the RF model and model performance. Download FIG S2, TIF file, 0.8 MB. Copyright © 2019 Sun et al. 2019 Sun et al. This content is distributed under the terms of the Creative Commons Attribution 4.0 International license.

**FIG 3:**
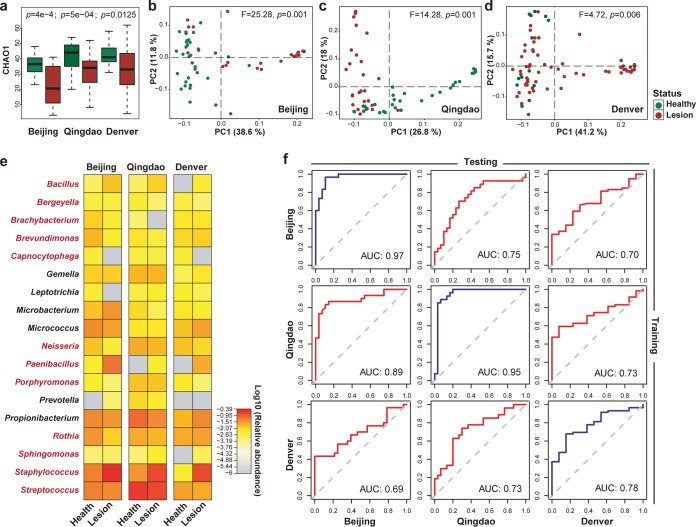
City-specific diagnosis models for AD. (a to d) The α diversity (a) and β diversity (b to d) of skin microbiota was significantly changed between healthy and lesional samples in each of the three cities. (e) Heatmap showing the relative abundances of 18 shared genera for building the city-specific AD diagnosis models. Among the three cities, the change patterns between healthy and diseased samples were consistent for 12 genera, which are highlighted with red font. (f) Performance of cross-city prediction using each city-specific AD diagnosis model, as assessed via the area under the ROC curve (AUROC). The ROC curve of 10-fold cross-validation was marked as blue lines and the ROC curve of the prediction as red lines.

To test whether differences in skin microbiome in AD were consistent by city, we compared lesional to healthy samples within each city. Reduction in α diversity was associated with AD in each city (Fig. 3a; *P = *0.0004 for Beijing; *P = *0.0005 for Qingdao; and *P = *0.0125 for Denver; Wilcoxon test, Chao1 index [41]). The β diversity was also distinct between healthy and AD microbiota (Fig. 3b to d; F = 25.28, *P = *0.001 for Beijing; F = 14.28, *P = *0.001 for Qingdao; and F = 4.72, *P = *0.006 for Denver; Adonis) in each city, suggesting the feasibility of microbiome-based diagnosis. RF models built for each city achieved diagnosis accuracy of 91.3% (area under the concentration-time curve [AUC], 0.97 for Beijing; Fig. S2b), 89.4% (AUC, 0.95 for Qingdao; Fig. S2c), and 79.2% (AUC, 0.78 for Denver; Fig. S2d) by 10-fold cross-validation, respectively. Underlying the power were 48, 28, and 33 marker genera, respectively, selected based on the rank order of variable importance. Of these, 18 genera were shared across cities (Fig. 3e and S3). Notably, *Staphylococcus* species were a common AD-associated (positively) genus among the cities, based on univariate statistical analysis (*P = *0.012, Wilcoxon rank-sum test). Training a diagnosis model in one city and applying it to another led to lower, yet still meaningful, accuracy (Fig. 3f); for example, application of the Beijing model (i.e., the Beijing cohort as training data) on Qingdao or Denver data sets would result in a reduction of AUC (10-fold cross-validation) from 0.97 to 0.75 and 0.70, respectively. Similarly, application of the Qingdao model (i.e., the Qingdao cohort as training data) on Beijing or Denver data sets resulted in a reduction of AUC (10-fold cross-validation) from 0.95 to 0.89 and 0.73, respectively. Application of the Denver model (i.e., the Denver cohort as training data) on Qingdao or Denver data sets resulted in a reduction of AUC (10-fold cross-validation) from 0.95 to 0.89 and 0.73, respectively.

10.1128/mSystems.00293-19.3 FIG S3 Bacterial genera that are differentially distributed between healthy and lesion samples pooled from the three cities of Beijing, Qingdao, and Denver. Download FIG S3, TIF file, 0.6 MB. Copyright © 2019 Sun et al. 2019 Sun et al. This content is distributed under the terms of the Creative Commons Attribution 4.0 International license.

### An AD diagnosis model applicable for all the three cities.

**FIG 4:**
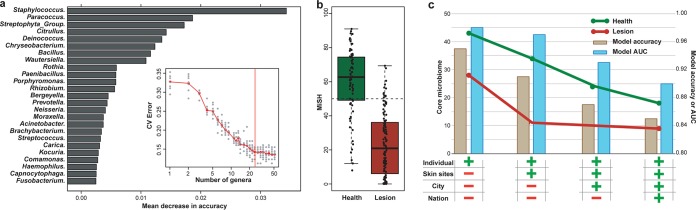
A universal model for AD diagnosis via skin microbiome. (a) The 25 genera with the most discriminating power in the universal diagnosis model were selected as disease markers. The bar length at each row indicates relative contribution of the genus to the RF model. Inset, relationship between the number of variables (i.e., genera) in the RF model and model performance. (b) MiSH distinguishes healthy and lesional samples. (c) Correlation between core microbiome size and model performance along the spatial scaling of geographical distance. The *y* coordinate (line plot) depicts the size of core microbiome. The *x* coordinate (bar plot) describes model accuracy (i.e., AUC). The bottom table describes the spatial scales of sampling, with “+” and “−” indicating inclusion and exclusion of samples, respectively, at each of the scales.

To test the feasibility of a generally applicable AD diagnosis model, we built an RF model using all the lesional and healthy samples from all three cities, using profiles of the taxa at six different phylogenetic levels (from genus to phylum; see Materials and Methods). The AUC was maximized at the genus level, and performance improvement was minimal when the top 25 most discriminatory genera were included (Fig. 4a, inset). We chose 25 genera, Staphylococcus, Paracoccus, Streptophyta, Citrullus, Deinococcus, Chryseobacterium, Bacillus, Wautersiella, Rothia, Paenibacillus, Porphyromonas, Rhizobium, Bergeyella, Prevotella, Neisseria, Moraxella, Acinetobacter, Brachybacterium, Streptococcus, Carica, Kocuria, Comamonas, Haemophilus, Capnocytophaga, and Fusobacterium as AD markers (Fig. 4a), and constructed a metric called Microbial Indicator of Skin Health (MiSH) that ranges from 0 to 100 for clinical diagnosis of AD, by multiplying the probability of being healthy in the RF model by 100 for each sample (see Materials and Methods). The MiSH model reached 86.4% diagnosis accuracy for all healthy and lesional samples combined from the three cities (AUC, 0.90; Fig. 4b), despite population and methodological differences among studies.

To test its spatial scalability, performance of MiSH was compared among (i) matching sites of individuals in a city, (ii) among individuals in a city, (iii) among individuals from the two Chinese cities, and finally (iv) among individuals from all the three cities (Fig. 4c). A stepwise reduction of diagnostic accuracy (AUC) from 0.98, 0.97, 0.93, to 0.90 suggests that introduction of microbiota heterogeneity at each additional spatial scale would reduce model performance. Consistent with this, for healthy samples, the size of the core microbiome (defined as the number of genera found in >50% of samples) followed a similar downward trend; this indicated an effect based on number of accumulated samples rather than geography, because reduction of the core microbiome size was correlated with the number of samples rather than the number of cities. For lesional samples, the core microbiome size largely plateaued when extending the scale beyond a single city and was smaller than the core microbiome of the healthy samples at all scales (Fig. 4c), confirming a conserved set of AD markers that is largely independent of the city of origin. This observation explains why this diagnosis model scales over large geographic distances.

### A suboptimal health state of nonlesional skin sites is confirmed using MiSH.

**FIG 5:**
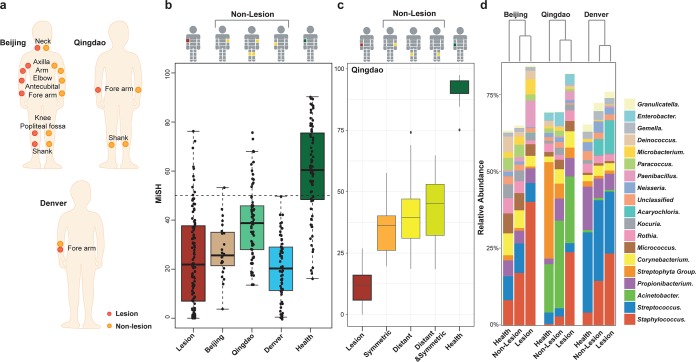
Definition and detection of the suboptimal health state of skin via skin microbiota. (a) Map of nonlesional samples on the skin. Beijing cohort, sites symmetrical to the lesional sites; Qingdao cohort, the symmetric sites of the lesional sites, plus another two remote sites on forearm or shank; Denver cohort, the surrounding, lesion-free skin of the lesional sites. (b) MiSH of nonlesional samples in the three cities compared to those of healthy and lesional samples. (c) MiSH of Qingdao nonlesional samples, which include one symmetric site (arm; “symmetric”) and two remote sites (leg; “distant” and “distant & symmetric”). (d) Profiles of the dominant genera (average relative abundance, >1%) in healthy, nonlesional, and lesional samples. In each of the three cities, the nonlesional microbiota is more similar to lesional ones than to healthy ones.

We next derived the MiSH for the lesion-free samples widely distributed on body surface of AD-active children, as follows (Fig. 5a): sites symmetric to the lesional sites (Beijing cohort), at three sites on the body (Qingdao cohort), and ∼5 cm away from the edge of the lesional site (Denver cohort). A vast majority of these nonlesional samples on AD-active children (95.8%, 78.6%, and 98.3% for Beijing, Qingdao, and Denver, respectively) carried a MiSH between 20 and 75. Their MiSH values were closer to those of lesional skins than those of healthy samples (Fig. 5b), although differences between lesional and healthy samples were still found in each city (paired *t* test; *P* = 6.2e−9, 1.8e−19, and 3.0e−10, respectively). For the Qingdao cohort, nonlesional sites included three different skin locations (forearm, left shank, and right shank) for each of the 29 AD patients (Table S1). Among the three sites, MiSH exhibited a pattern of recovering with increasing distance to the lesional sites (Fig. 5c); however, alteration of the skin microbiota at a nonlesional site was not strongly correlated with its distance from the lesional site.

Interestingly, microbiome structures of nonlesional sites differed from both healthy and lesional sites, although the nonlesional microbiota are more similar to lesional than to healthy sites in each of the cities (Fig. 5d). Thus, nonlesional samples feature dysbiosis of the microbiota, which has already shifted toward the diseased state, even though both the healthy states and the diseased states differ in each city. Notably, this observation in pediatric cohorts is consistent with previous observation of dysbiosis of microbiome on nonlesional skin sites in adult AD patients (7, 42). In particular, S. aureus was more abundant in the nonlesional samples than in healthy samples (*P < *0.01, Wilcoxon test), yet it was less abundant than in lesional samples (*P < *0.01, Wilcoxon test). The higher susceptibility to S. aureus colonization suggests these nonlesional samples as a risk-prone state of skin between healthy and the diseased, i.e., a “suboptimal health” (SoH) state. Importantly, the SoH state was not limited to the immediately surrounding or adjacent area of AD-active zones but occurs across the whole body surface (Fig. 5b and c). It has indeed been observed that S. aureus colonization or microbiome dysbiosis can precede AD in early childhood (43, 44).

### Assessing AD treatment via the skin microbiome using MiSH.

**FIG 6:**
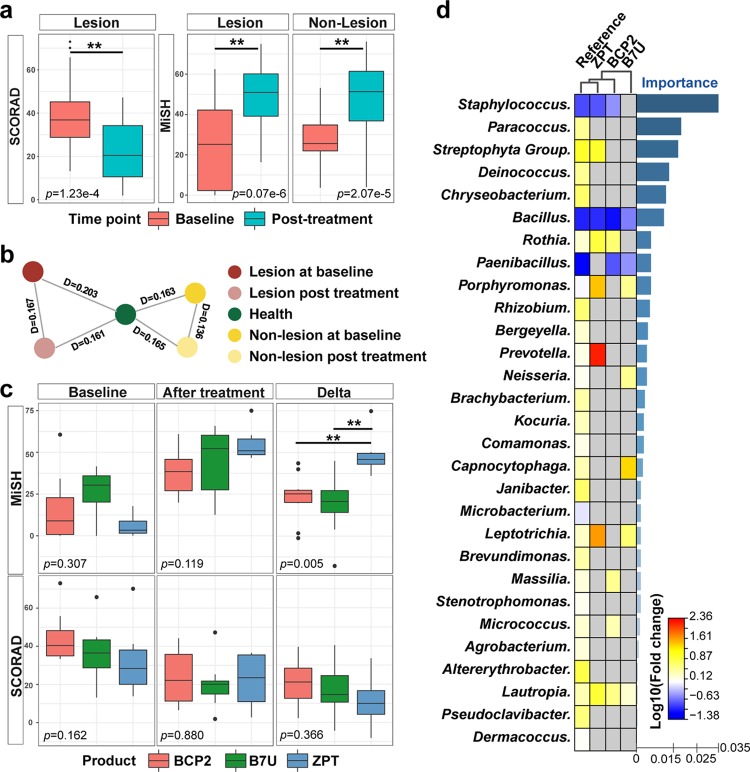
Assessing and comparing the efficacy of skin care products via skin microbiota. (a) Corticosteroid treatment induced change of SCORAD (left) and MiSH (middle and right) in both lesional and nonlesional sites. (b) Change in skin microbiota due to the treatment. (c) Boxplots of MiSH and SCORAD before and after the treatment via each of the three active ingredients in body wash soap. Boxes represent the interquartile range (IQR), and the lines inside represent the median. Whiskers denote the lowest and highest values within 1.5× the IQR. ΔmiSH, posttreatment subtracted by baseline; ΔSCORAD, baseline subtracted by posttreatment. (d) Characteristic change pattern in skin microbiota between posttreatment and baseline under each of the three active ingredients in body wash soap. Change in relative abundance of bacterial markers, sorted by importance in the diagnosis model, was shown as a heatmap. For the “Reference” pattern, which is between healthy and lesional samples and thus represents the change corresponding to a full recovery, the relative abundances of 36 bacterial genera changed significantly, and the 29 shared among the three cities are shown. Among the three active ingredients, ZPT carries a microbiota change pattern the most similar to “Reference,” suggesting its higher efficacy than BCP-2 or B7U in inducing recovery of skin microbiota back to the healthy state.

We next asked whether treatment of AD is associated with recovery of skin microbiota at the lesional and nonlesional sites. For each of the AD-active children in Beijing, an identical dose of corticosteroid was applied to the lesional sites every day for 4 weeks (see Materials and Methods). The SCORAD significantly improved (*P = *1.23e−4; Student’s *t* test, Fig. 6a). On the other hand, MiSH of both lesional and nonlesional samples, at 20.2 ± 17.9 and 25.1 ± 10.4, respectively, before treatment, both significantly improved after the treatment (41.3 ± 16.7, *P = *9.07e−6 and 40.9 ± 19.9, *P = *2.07e−5, respectively; Student's *t* test; the responses at lesional and nonlesional sites were statistically indistinguishable; *P = *0.43; Fig. 6a). Moreover, the treatment moved the skin microbiota of both lesional and nonlesional sites across the body to a structure more similar to that of healthy samples than to lesional or nonlesional ones (Fig. 6b).

These results illustrated the possibility of using MiSH for objectively assessing the potential efficacy of treatment regimens, which would be a significant advance because a change in host phenotype can be difficult to quantify (15, 45). To test this hypothesis, for Beijing cohort, three kinds of soap containing different active ingredients (BCP-2, B7U, or zinc pyrithione [ZPT]) were used for body wash (once daily) along with local application of corticosteroid on AD-active children for 4 weeks (see Materials and Methods). At baseline (or posttreatment), no difference in MiSH or SCORAD was apparent among the three treatment groups, yet the MiSH was elevated and SCORAD reduced after each of the three treatments (Fig. 6c). Notably, ΔMiSH, the difference between baseline and posttreatment that quantifies the degree of AD recovery, was much higher for the ZPT group (ΔMiSH = 49.2 ± 12.2; *P = *0.001 and 0.002, Student's *t* test) than the other two (ΔMiSH = 22.9 ± 13.4 for BCP-2; ΔMiSH = 18.7 ± 17.9 for B7U), suggesting higher effect of ZPT on remediating skin microbiota in AD. Although no difference in clinical efficacy was detected based on SCORAD (Fig. 6c), our findings raised the possibility that shift in MiSH can be used to assess the efficacy of skin care products via the microbial diversity change of skin. Rational validations of such findings in the larger human population could eventually lead to the novel prognosis strategy for AD-inflicted individuals.

A comparison of microbiome changes for the three treatments explains their differential influence (Fig. 6d). The change that distinguishes healthy from lesional samples, e.g., the significant decrease in Staphylococcus, Bacillus, and Paenibacillus spp., was designated a “reference” that presumably corresponds to a full recovery from AD. ZPT induced a microbiota change pattern the most similar to the reference; the relative abundance change of eight taxa (the top three being *Staphylococcus*, *Bacillus,* and Streptophyta) after the 4-week treatment is consistent with the reference (Fig. 6d). Such superior efficacy of ZPT is likely due to its antibacterial activity (neither BCP-2 nor B7U contains antibacterials), which kills more AD-associated bacteria (in the reference), such as S. aureus, and thus shapes skin microbiota to a healthier state (46). PCoA of samples before and after usage of the skin care products suggested that the microbiota after ZPT treatment was more similar to that of healthy ones than the other two treatments (Fig. S4), consistent with those from the heat map (Fig. 6d). Therefore, the change in skin microbiome appears to be sufficiently sensitive to characterize and evaluate the effects of ingredients in body wash soap, and such microbiome signatures may form a basis for assessing and comparing treatment efficacy on skin microbiome.

10.1128/mSystems.00293-19.4 FIG S4 PCoA of samples before and after usage of the skin care products. Download FIG S4, TIF file, 0.3 MB. Copyright © 2019 Sun et al. 2019 Sun et al. This content is distributed under the terms of the Creative Commons Attribution 4.0 International license.

### MiSH stratifies AD patients and predicts their response to skin care treatment.

**FIG 7:**
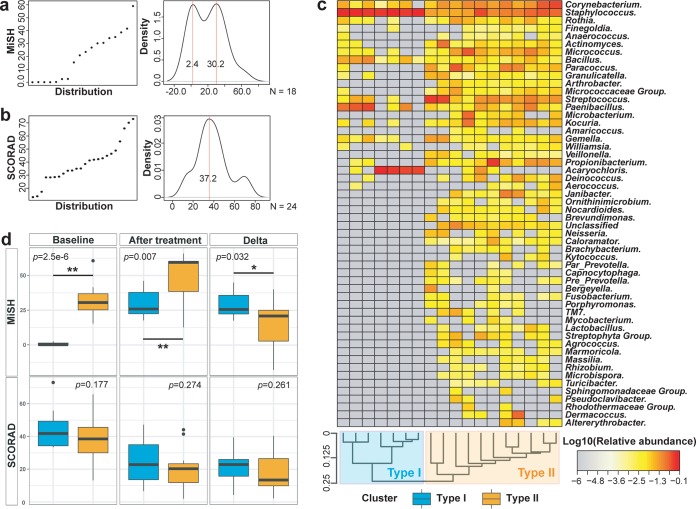
Predicting the host response to treatment via skin microbiota. (a and b) Distribution (left) and density (right) of MiSH (a) and SCORAD (b) in the 18 patients at baseline. The MiSH, but not SCORAD, exhibits a multimodal (bimodal) distribution, suggesting patient stratification into distinct disease state prior to treatment, despite their equivalent SCORAD. (c) AD-active children were classified based on their baseline skin microbiota into types I and II, with their organismal signatures shown as a heatmap. (d) Pre- and posttreatment MiSH for the types I and II of patients. The SCORAD index is also shown for comparison purposes.

Interestingly, the baseline MiSH (but not the baseline SCORAD) from lesional samples of the 18 patients in the BCP2 and B7U treatment groups exhibited a bimodal distribution (Fig. 7a and b; BCP2 and B7U induced equivalent improvement in MiSH; the effect of ZPT was distinct; thus, ZPT was excluded from this test). Thus, despite their equivalent SCORAD, the 18 patients can be stratified at the baseline (i.e., before any treatment) via MiSH into two types of distinct disease states, type I of seven patients and type II of 11 patients. Hierarchical clustering of the pretreatment lesional microbiomes from AD patients generated two classes that exactly correspond to the type I and II hosts defined above, supporting the microbiome basis for this stratification (Fig. 7c). Type I features significantly fewer genera (54 versus 20, stats) but a much higher proportion of *Staphylococcus* spp. (3.9 times of type II, stats). In contrast, type II features higher relative abundance of 20 genera (from the phyla of *Actinobacteria, Firmicutes, Proteobacteria, Thermi, Bacteroidetes, Fusobacteria*, and *TM7*). The removal of *Staphylococcus* before rarefaction led to similar classification, confirming the importance of these genera as markers of the two types.

The baseline MiSH for type I patients (MiSH = 0.9 ± 1.1) were much lower than those of type II (MiSH = 32.5 ± 11.5; *P = *2.5e−6, Student's *t* test; Fig. 7d and Table 2), which indicates that type I patients carried a more disease-oriented microbiome. Posttreatment MiSH for type I patients were also significantly lower than those of type II (30.0 ± 10.0 and 48.4 ± 16.0, respectively; *P = *0.007; Fig. 7d); thus, type II patients recovered to a microbial state more closely resembled healthy skin. However, the ΔMiSH for type I patients were significantly higher than type II (29.2 ± 10.1 versus 15.9 ± 16.5, *P = *0.032; Fig. 7d), suggesting a more prominent response of microbial diversity recovery for type I patients; this is likely due to the much lower MiSH for type I patients at baseline. In contrast, none of baseline SCORAD, posttreatment SCORAD, or ΔSCORAD during treatment were different between type I and type II (Fig. 7d). Notably, patients with identical SCORAD were not necessarily of identical MiSH because SCORAD depicts the physiological change of AD patients, while MiSH depicts the changes of skin microbiota. Besides, corticosteroids therapy may affect the correlation between skin microbiota and SCORAD during AD recovery. Collectively, these results demonstrate prognosis of treatment response via MiSH prior to treatment, as type I patients, who are defined by a worse form of diseased microbiome, tend to undergo a greater recovery during the treatments.

**TABLE 2:** MiSH and SCORAD of the 18 AD patients in the BCP2 and B7U treatment groups, both prior to treatment and posttreatment

Sample ID	Type	MiSH at:		SCORAD at:	
		Baseline	After treatment	Baseline	After treatment
1039	I	0.06	17.64	33.40	6.62
1069	I	0.14	19.88	55.80	36.50
1072	I	0.10	25.06	33.57	10.65
1052	I	0.22	25.84	35.12	22.82
1050	I	2.60	30.62	73.00	33.50
1001	I	2.48	46.08	41.83	16.52
1024	I	0.02	45.06	42.91	47.24
1056	II	30.54	12.66	48.81	41.48
1080	II	60.62	59.42	28.30	10.35
1089	II	34.20	35.88	28.88	20.31
1005	II	26.90	30.84	65.75	25.29
1070	II	41.58	62.14	13.20	1.95
1030	II	38.68	59.60	46.39	44.14
1060	II	35.04	59.46	44.82	20.62
1064	II	15.38	41.04	38.55	7.79
1077	II	30.16	65.84	31.20	19.94
1078	II	20.90	60.92	42.24	13.31

### A MiSH-based scale for assessing and prognosing skin health in children.

**FIG 8:**
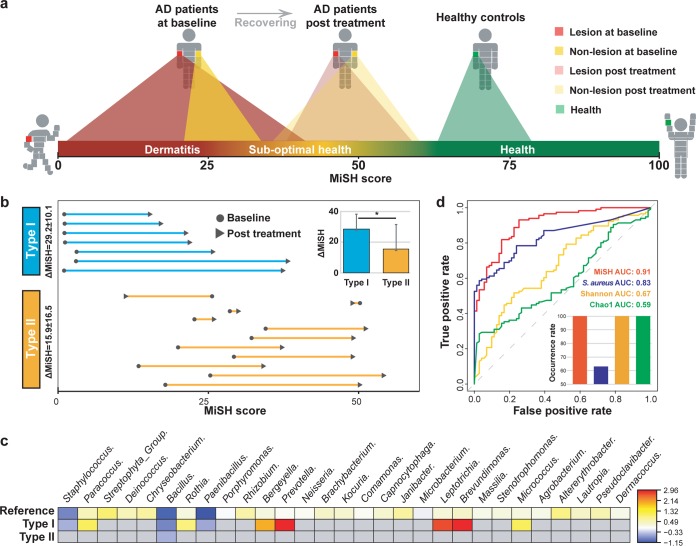
A universal scale to quantitatively assess and compare skin-health state via skin microbiota. (a) The scale of MiSH, ranging from 0 to 100, represents the probability of being healthy (i.e., from 0% to 100%). Color-shaded triangular areas represent the interquartile range (IQR) of the MiSH score. Notably, the MiSH of nonlesional sites are harbored within those of lesional sites, suggesting a diseased microbiota and a risk-prone state (i.e., the “suboptimal health”) of skin at the nonlesional sites. After the treatments, the MiSH of both lesional and nonlesional sites, despite significant improvement, stayed at a state that can be considered the suboptimal health, since its MiSH is still much lower than that of healthy children. (b) Changed patterns of MiSH for the two patient types, which indicate distinct baseline disease severity and different sensitivity to treatment. (c) Characteristic change pattern of skin microbiota between type I and type II under corticosteroids. (d) Comparing the performance of MiSH, S. aureus, Chao1, and Shannon index for AD diagnosis. The occurrence rate of S. aureus is shown in the inset.

We propose that, via MiSH and lesional status, a skin sample can be classified into one of three healthy states (Fig. 8a), as follows: (i) MiSH of <50 plus lesional, AD-active (i.e., <50% probability of being healthy, based on the diagnosis model); (ii) MiSH of <50 yet without lesion, SoH, i.e., a state that is distinct from either healthy or diseased states and has already shifted toward disease; and (iii) MiSH of >50, healthy (i.e., 50% probability of being healthy). Projection of microbiota and their metadata onto the common scale of MiSH underscores the potential of skin microbiota for personalized skin care (Fig. 8b). First, healthy hosts can be distinguished from AD-active hosts. Second, within AD-active hosts, nonlesional sites can carry a microbiome similar to that of lesional sites. Third, during treatment, the microbiota of both lesional and nonlesional sites moves toward the healthy microbiota, although a full recovery would take much longer than the treatment period. Finally, the patients can be classified into types I and II with distinct microbial diversity at their first visit, and different treatment effects (by corticosteroid) can be predicted (Fig. 8c).

At present, SCORAD is the prevalent clinical metric for AD diagnosis, yet it is limited by subjectivity in judgment and inability to evaluate risk-prone states of skin that exhibits no visible symptoms. Microbial α diversity is also linked to skin health (39), yet the performance of Shannon index or Chao1 index for AD diagnosis is poor (AUC, 0.67 and 0.59 separately; Fig. 8d), as no clear decision boundary can be obtained from either metric (Fig. S5a). S. aureus, to a certain extent, is considered a biomarker of AD (48), as it was enriched in AD while barely detected in healthy children (49). Moreover, it was recently reported that among *Staphylococcus* spp., S. aureus predominates in more severe AD and S. epidermidis predominates in less severe AD (50). However, an AD diagnosis model based on S. aureus alone carries an AUC of 0.83, much lower than that of MiSH (0.90). This is due to the large interpersonal variation of S. aureus in AD patients (51); its occurrence rate can be rather low among all hosts (39% for healthy samples, 87% for lesions, and 63% overall; Fig. 8d) and varies widely among the Beijing, Qingdao, and Denver cohorts (81%, 26%, and 95%, respectively; Fig. S5b). Therefore, a reference range of S. aureus trained in one population would not apply in others, as the intercity variation in its relevant abundance is quite high, even for healthy children (*P = *0.012, Kruskal-Wallis test; Fig. S5c).

10.1128/mSystems.00293-19.5 FIG S5 Shannon index and the level of Staphylococcus aureus in pediatric skin microbiota from the three cities of Beijing, Qingdao, and Denver. (a) No significant difference was found in the Shannon index among the healthy, lesion, and nonlesion samples. Occurrence rate (b) and relative abundance (c) of S. aureus in each city are also shown. Download FIG S5, TIF file, 0.2 MB. Copyright © 2019 Sun et al. 2019 Sun et al. This content is distributed under the terms of the Creative Commons Attribution 4.0 International license.

In our study, MiSH is significantly correlated with SCORAD (Fig. S6a; rho = 0.46, *P = *6e−4, Pearson test) and with skin microbial α diversity (Fig. S6b; rho = 0.58, *P = *2e−16, Pearson test). MiSH is also positively correlated with the relative abundance of S. aureus (Fig. S6c; rho= 0.63, *P = *2e−16, Pearson test). In the Qingdao cohort, the S. aureus-dominated group and an S. epidermidis-dominated group (ratios of S. aureus and S. epidermidis were determined by quantitative PCR [qPCR]; see Materials and Methods) were of significantly different MiSH, with S. aureus carrying lower MiSH (indicating more severe AD; *P = *0.031). Thus, MiSH can be reconciled with existing biomarkers of AD and can potentially serve as a clinically useful and generally applicable parameter.

10.1128/mSystems.00293-19.6 FIG S6 Link between MiSH and SCORAD, Chao1, relative abundance of S. aureus, or body site. (a) Correlation between MiSH and SCORAD. (b) Correlation between MiSH with the Chao1 index. (c) A high Spearman coefficient was found between MiSH and relative abundance of S. aureus. (d) MiSH is applicable to the various body sites, which were sampled in the Beijing cohort. Tenfold cross-validation was performed. (e) Distribution of MiSH in the Beijing, Qingdao, and Denver cohorts. Download FIG S6, TIF file, 0.5 MB. Copyright © 2019 Sun et al. 2019 Sun et al. This content is distributed under the terms of the Creative Commons Attribution 4.0 International license.

These advantages of MiSH encourage us to establish an interactive website which accepts 16S rRNA-amplicon based data sets as input and returns a graphical report of MiSH (http://bioinfo.single-cell.cn/mish/index.php/upload_mish) (see Materials and Methods). This online tool may be of potential value in personalized skin health assessment, prediction of response to treatment, and comparison of skin care product effects in both healthy and AD-active children.

## DISCUSSION

Enthusiasm for diagnosis and therapy of skin disorders via skin microbiota has arisen from recent evidence that: (i) the dysbiosis of skin microbiota is not just associated with skin inflammations (39) but can be a driving factor (52), (ii) S. aureus colonization precedes the onset of AD in certain children (41, 44), (iii) commensal skin bacteria protect against pathogens (7), and (iv) recovery of skin health should require restoration of the healthy microbiota (15). However, these studies are mostly performed in a single localized and relatively homogenous cohort. However, in addition to body region and host individuality (53), the main factors affecting the skin microbiota include geographical location (54, 55). The lack of understanding of variation between healthy and diseased skin microbiota scales at different spatial dimensions, i.e., among geographically separated populations, has hindered critical assessment and exploitation of the potential of the skin microbiota as a quantitative, objective, and widely applicable barometer for skin health. Notably, the difficulty in transplanting microbiome-based disease models between populations is common; for example, the utility of intestinal microbiota in diagnosis is hindered by an effect size of individual hosts that is larger than that of disease status (e.g., lean and obese individuals assessed by the gut microbiota [56–58]).

A comparison of the skin microbiome of AD and healthy pediatric cohorts from three cities, two Chinese and one American, revealed that, healthy and diseased skin microbiota from each city carried both city-specific signature (Fig. 3e) and AD-associated biomarkers (Fig. 4a), and there were significant overlaps among the healthy and diseased biomarkers. Although the two Chinese cities shared more disease biomarkers than did the intercontinental pairs, a significant core of AD-associated microbiota was present, with its size and membership independent of geographic distances among populations. Therefore, despite the differences among pediatric cohorts, an AD diagnosis model built from a single city can be applied across the three cities with acceptable accuracy. As a result, despite the large effect size of city and individual variation, the MiSH model consisting of the top 25 bacterial skin genera can diagnose AD with 86.4% accuracy (AUC, 0.90) across cities and continents, and it offers high sensitivity in assessing the efficacy of treatment products. Notably, although the body location is one of the most important factors to the skin microbiome (54, 55), application of MiSH (which was generated based on all samples from the three cities) on the Beijing samples of various body locations revealed that, in each of the six body locations that include both moist (antecubital fossa and popliteal fossa) and dry (arm, knee, neck, and shank) ones, MiSH can reliably distinguish their health status (Fig. S6d).

Moreover, for nonlesional skin sites in AD-active children, the MiSH model revealed a distinct state of skin microbiota called suboptimal health, which is intermediate between those of lesional sites and healthy children, yet more similar to the former, and carries a level of *Staphylococcus* spp. higher than that in healthy hosts but lower than in lesional sites. This state was converted to a healthier state on the MiSH scale after topical medication. However, the degree of dysbiosis or its remediation does not correlate with physical distance to the lesional site. Although initial evidence for the alteration of microbiota on apparent healthy skin zones physically adjacent to the lesional sites has emerged (59, 60), the extent to which the skin microbiota respond across the whole body is not known. Our findings here support AD as a topical effect but with an underpinning microbiota dysbiosis that extends across the body (61, 62), and they underscore the dynamic interactions between global host immune response and local skin microbiota (63). Therefore, MiSH can be used not only for AD severity measurement but also for assessing the healthy state and the risk-prone state of skin in AD-free children (whether this is applicable in adults is unknown, as AD skin microbiome is affected by age [64]).

Furthermore, pretreatment MiSH classifies children with clinically indistinguishable AD into two types with distinct disease severity and sensitivity to corticosteroid therapy. These two types of patients feature distinct microbiota structures prior to treatment and exhibit characteristic patterns of microbiota change during treatment. Type I patients, with lower MiSH at baseline, carry a more disease-oriented microbiome that features fewer genera yet much higher proportion of *Staphylococcus* spp., represent a more severe AD form, and tend to have a more prominent response of recovery during treatment. In contrast, type II patients, with higher MiSH at baseline, carry a less disease-oriented microbiome characterized by a lower level of *Staphylococcus* spp. yet higher diversity of bacterial genera and represent a milder disease form of AD. Interestingly, in Qingdao and Denver, the MiSH of all AD patients also exhibit a bimodal distribution (Fig. S6e; our current data do not allow testing of whether such clustering is correlated with treatment effect in these two cities). Accordingly, the two types should be treated differently; for example, type I should be prioritized for treatment with higher drug dosage, since it represents a more severe form of AD yet is more likely to respond to treatment in terms of skin microbiota recovery. Consistent with a recent study that suggests cross-modulation of the skin microbiome, skin surface microenvironment and immune system underlie susceptibility to AD in adults (42), our findings here support a microbial basis for the heterogeneity of response to AD treatment and for the recovery of skin health in children. Notably, two distinct clusters of skin microbiome were discovered in the lesion samples from 51 adult psoriasis patients from New York City (65), although whether their disease outcomes or treatment effects are different remains to be tested. Therefore, it seems possible that such microbiome-defined cutaneotypes can be quite common in disease, and further characterization of cutaneotypes within and across various kinds of skin inflammations might provide new insights into disease diagnosis or treatment strategy.

At present, one limitation of MiSH is its inability to distinguish the various *Staphylococcus* species due to the genus-level resolution of 16S rRNA amplicon-based sequencing in microbial identification. Recent reports suggested that different *Staphylococcus* species can play distinct roles in AD development; for example, S. epidermidis and Staphylococcus hominis, which predominantly reside on healthy human skin, actually contribute to cutaneous homeostasis and health (8); in addition, selected *Staphylococcus* strains can either promote cutaneous antimicrobial activity or trigger inflammation in AD (7, 50). Thus, versions of MiSH that assess skin microbiota at the species or the strain levels should be developed, via either long-read sequencing of 16S rRNA amplicons or metagenome sequencing. Moreover, tools such as conditionally rare taxa (CRT) (66) can be used to probe the scope and origin of such city-specific bacterial taxa, as they offered 97.6% to ∼100% accuracy in distinguishing the three cities and a level of performance in distinguishing the AD status that is equivalent to that with MiSH (Materials and Methods).

On the other hand, as size of the treatment cohort here is relatively small, how generally applicable the microbiome-defined heterogeneity in treatment response is not yet clear, and its mechanism is unknown. Future efforts tackling these questions are key to more precise AD therapies (67, 68). Despite these limitations, once the costs of sequencing are reduced to an acceptable level, MiSH is expected to contribute, in conjunction with SCORAD, for AD diagnosis and treatment in the clinical setting, where the state of skin microbiota is also taken into consideration.

## MATERIALS AND METHODS

### Study design.

From the city of Beijing, China (the Beijing cohort), we established a cohort of 28 children age 4 to 12 years who were suffering from mild to moderate AD, plus 30 age-equivalent and sampling site-matched children with no personal or family history of AD and no history of chronic skin or systemic diseases (Table S1). To explore the link between AD treatment and skin microbiota alteration, an “AD-treatment cohort” was designed, in which AD-active children of the Beijing cohort underwent a 4-week-long treatment regimen of corticosteroid administration, with each child using one of the three skin care products of BCP2 (ultramild body wash with lipids), B7U (mild synthetic bar) or ZPT (ultramild body wash with lipids and zinc pyrithione).

In addition, a second cohort of age-matched children was recruited from Qingdao (the Qingdao cohort), a coastal Chinese city at the West Pacific rim 650 kilometers southeast of Beijing. The Qingdao cohort consists of 29 pediatric patients suffering from moderate AD and 30 healthy subjects, who were screened using selection criteria that are identical to those for the Beijing cohort.

Furthermore, to test whether healthy and AD-active skin microbiota patterns held true at even greater geographic distances, a third cohort we previously published for Denver, an inland city of North America (the Denver cohort), was also included into the three-way, cross-city comparison here. The cohort consists of 59 AD-active and 13 healthy children that were 4 to 12 years old (60). Similar to this work, the Denver study employed MiSeq paired-end reads and the primer set of 27F/534R for profiling bacterial 16S rRNA amplicons (Table S3). Moreover, to ensure data comparability, samples from the three cities were computationally processed in an identical manner.

10.1128/mSystems.00293-19.10 TABLE S3 Comparison of experimental protocols for skin microbiome sequencing among the three cities. Download Table S3, DOCX file, 0.02 MB. Copyright © 2019 Sun et al. 2019 Sun et al. This content is distributed under the terms of the Creative Commons Attribution 4.0 International license.

Exclusion criteria for all subjects include having a fever of ≥38.5°C, having bathed or showered after midnight before the day of sampling, using creams/lotions at the sites 24 h prior to sampling, having received oral antibiotics, a bleach bath, or topical prescription medications (including but not limited to Elidel, Protopic, topical corticosteroids, or topical antibiotics; more details below) 7 days prior to sampling, having taken systemic immunosuppressive drugs (including cyclosporine or oral steroids), and having experienced total body phototherapy (e.g., UV light B, psoralen plus UV light A, and tanning beds) within 20 days prior to sampling.

The study was conducted and all samples were collected with approval from the Procter & Gamble Beijing Innovation Center institutional review board and in accordance with the World Medical Association Declaration of Helsinki (1996 amendment). ICH Guidelines for Good Clinical Practice (GCPs) were followed, and voluntary informed consent was provided with the approval of the Research Ethics Board of P&G. Mothers who agreed to have their children participate in this study signed an informed consent form, and teenagers who agreed to participate signed an assent form.

### Severity scoring of atopic dermatitis.

Only subjects in the AD group would undergo dermatologic evaluations throughout the study. Subjects acclimated for a minimum of 30 minutes in an environmentally controlled room (maintained at 70°F and 30 to 45% relative humidity) prior to undergoing a dermatologic evaluation from the study dermatologist at the following time points. At the baseline and week 4 visits, the dermatologist would assess the subject’s atopic dermatitis lesional/measurement sites on their arms and/or legs only for the intensity of objective attributes. This evaluation along with the extent of body surface involvement and subjective symptoms (pruritus and sleep loss) rated by the subject was used to determine the SCORAD value (22). The SCORAD value is calculated using the following formula:

$$\text{SCORAD}=\frac{\text{Extent}}{5}+\frac{7\times \text{Intensity}}{2}+\text{Subjective signs}$$

where (i) “Extent” is the extent of body area affected; to determine the extent of affected area as a percentage of the whole body, the rule of nine is used. (ii) “Intensity” is the intensity grading scale; the marked lesional sites are graded for the intensity of each of the following signs: dryness, erythema, excoriation, weeping, induration, and lichenification. (iii) “Subjective signs” is the subjective symptoms, where itch and sleeplessness are each scored by the subjects or parent/guardian using a 10-cm visual analogue scale where 0 is no itch (or no sleeplessness) and 10 is severe itch (or sleeplessness).

### Skin microbiome sampling strategy.

In each of the three cohorts, microbiota from skin zones corresponding to the AD-active sites of patients were sampled in matched healthy individuals. Additionally, for each AD-active child, skin microbiota from both lesional and nonlesional sites was collected. For the Beijing and Denver cohorts, nonlesional sites were taken from nonlesional skin site of a symmetric location on the body or the surrounding skin of the lesional sites, and for the Qingdao cohort, the nonlesional sites also included another two sites on the forearm and shank surface (Table S1).

At the inclusion visit and at the end of study, the same investigating dermatologist evaluated the children via the SCORAD (SCORing Atopic Dermatitis) index, which is a clinical tool for assessing AD severity (22). Only individuals with a SCORAD index between 25 and 40 at baseline were included as patients in the study. Skin microbiota samples of lesional skin were collected using aseptic techniques under sterile airﬂow generated by a portable hood. Similarly, samples were also collected from the unaffected symmetric and remote body skin area.

Sampling procedures were as follows. (i) Identify the designated sampling site being used for swab collection (≥10 cm^2^) and then use a ruler/template to mark an 8-cm^2^ area. (ii) Identify the designated nonlesion site being used for swab collection. (iii) Label all collection tubes. (iv) With gloved hands, remove DNA swab from packaging with care taken not to touch any surface. (v) Dip the swab tip into NaCl plus Tween 20 solution, and press the swab to the inside of the tube to remove any excess liquid. (vi) Apply the swab in both horizontal and vertical directions (totally 50 times, for about 30 to ∼35 s) for sampling the marked area. (vii) Break the DNA extraction swab and put into the appropriately labeled empty 2-ml tube and cap. (viii) Store the tube in an ice box until samples can be stored at –80°C. Finally, repeat steps iv through viii on a nonlesion site for each site.

### Administration of medication for AD treatment.

In the city of Beijing, for the 28 AD patients who were sampled from both lesional skin sites and nonlesional sites at baseline and then again after 4 weeks of treatment via corticosteroids and bath products, only 24 of the patients were evaluated posttreatment because four individuals failed to show up for the last visit. The treatment was via corticosteroid (0.1% hydrocortisone butyrate ointment), which was used on every patient based on doctor’s advice. In addition, one of three body wash products was used in bath, BCP2, ZPT, or B7U. Treatment assignment was randomized to subject to balance for baseline AD severity, age, biological sex, and body location if possible. Due to the complexity and smaller sample sizes, the balancing was prioritized in order of importance, with baseline disease severity, age, biological sex, and then body location (most of the subjects had lesions on arms). Patients were instructed to apply the bath product once daily in the evening to their entire body. Patients were also asked not to change their hygiene practices or to apply any other bath products during the study.

Specifically, this is a 7-week, randomized, double-blind, parallel group in-home-use study among male and female subjects who are 4 to 12 years of age (inclusive) and having mild to moderate active atopic dermatitis (AD), where three products were tested. Written informed consents were obtained from the parent/legal guardian of each subject and verbal assent from each subject according to ICH GCPs prior to screening based on the inclusion/exclusion criteria listed below. Qualified subjects (including the healthy control group) completed a habits and practices questionnaire prior to starting the preconditioning phase. They completed a 7-day preconditioning phase where they used a provided bar soap for all body cleansing purposes and refrained from using any other personal cleansing products as well as any moisturizers, powders, topical medications, oils, or creams for the duration of the preconditioning phase. Subjects also refrained from using any body cleansing implements (e.g., wash cloths and body puffs) during the preconditioning phase of the study. Subjects were permitted to use their normal facial and hair care cleansing products, but they must refrain from using any products containing antibacterial ingredients (i.e., acne products, salicylic acid-containing facial care products, and antidandruff shampoos) during the preconditioning phase.

### Ingredients of the three body wash products tested.

The ingredients for B7U (regular synthesized bar soap) were sodium lauroyl isethionate, paraffin, sodium cocoglyceryl ether sulfonate, glycerin, water, talc, magnesium stearate, stearic acid, sodium isethionate, magnesium cocoate, sodium stearate, coconut acid, sodium chloride, sodium cocoate, fragrance/parfum, magnesium laurate, lauric acid, and titanium dioxide.

The ingredients for BCP2 (ultramild moisturizing body wash) were water, petrolatum, sodium trideceth sulfate, sodium chloride, cocamidopropyl betaine, trideceth-3, guar hydroxypropyltrimonium chloride, sodium benzoate, xanthan gum, glyceryl oleate, fragrance, disodium EDTA, citric acid, sodium hydroxide, acrylates/c10-30 alkyl acrylate cross-polymer, Butyrospermum parkii (shea) butter, methyl-chloroisothiazolinone, and methylisothiazolinone.

The ingredients for ZPT (0.5% zinc pyrithione containing ultramild moisturizing body wash) were water, petrolatum, sodium trideceth sulfate, sodium chloride, cocamidopropyl betaine, trideceth-3, zpt, guar hydroxypropyltrimonium chloride, sodium benzoate, xanthan gum, glyceryl oleate, fragrance, citric acid, sodium hydroxide, acrylates/c10-30 alkyl acrylate cross-polymer, Butyrospermum parkii (shea) butter, methylchloroisothiazolinone, and methylisothiazolinone.

### DNA extraction, PCR amplification, and sequencing of skin microbiome.

Genomic DNA was extracted from each swab using the Qiagen tissue and blood DNA isolation kit, following the manufacturer’s instructions, with slight modifications (69). PCR amplification of the V1-V3 region of 16S rRNA genes was performed using the primer set (27F/534R) and followed the protocol developed by the Human Microbiome Project. PCR amplification reaction mixtures in triplicate for each sample were pooled at approximately equal amounts and sequenced. For Qingdao and Denver, Illumina MiSeq was employed as the sequencing platform. For Beijing cohort, both MiSeq and Roche 454 FLX were used for sequencing each of the samples. Roche 454 sequencing data were used in building the RF model. MiSeq data were used to calculate the effect size of factors, so as to avoid the bias due to difference in sequencing platforms. For both healthy and lesional samples, the effect size of sequencing platform is smaller than that of city (Fig. S7). In addition, no positive PCR results were found in the negative controls (i.e., clean swabs), suggesting that no background bacterial contamination can be detected.

10.1128/mSystems.00293-19.7 FIG S7 Effect of sequencing platform on analysis of skin microbiota. Principal-component analysis was performed for all healthy and lesion samples from Beijing, Qingdao, and Denver. For the Beijing cohort, each sample was sequenced via both 454 and MiSeq; for the Qingdao and Denver cohorts, the samples were sequenced via MiSeq. (a) Healthy samples are highlighted with color, while lesion samples are in the background (gray). (b) Lesion samples are highlighted with color, while healthy samples are in the background (gray). For both healthy and lesion samples, the effect size of sequencing platform on skin microbiome (F = 5.63) is much smaller than AD status (F = 18.72) or city origin (F = 25.93). (c to f) Rarefaction curves of Roche 454 and Illumina MiSeq sequencing data. The curves of observed genera and Shannon index from Roche 454 (c and d) and from Illumina MiSeq (e and f) were shown. Download FIG S7, TIF file, 0.9 MB. Copyright © 2019 Sun et al. 2019 Sun et al. This content is distributed under the terms of the Creative Commons Attribution 4.0 International license.

For quantitative PCR (qPCR) that measures the relative abundance between S. aureus and S. epidermidis, the primer pair is 5′-TAGTTGTAGTTTCAAGTCTAAGTAGCTCAGC and 3′-ATTTAACCGTATCACCATCAATCG for S. aureus and 5′-GGCAAATTTGTGGGTCAAGA and 3′-TGGCTAATGGTTTGTCACCA) for S. epidermidis (70). Gene copy number was calculated based on the standard curve of each primer system using the LightCycler 480 software 1.5 (Roche). Relative abundance is defined as gene copy number of each biomarker divided by 16S rRNA gene copy number of whole bacteria. Each qPCR reaction was performed in triplicate.

### Sequence analyses of skin microbiomes.

All sequences were preprocessed following the standard QIIME (v.1.9) pipeline. A total of 643,038 high-quality partial 16S rRNA sequences were obtained from the 275 samples collected, with an average of 8,669 sequences per sample. Downstream bioinformatics analysis was performed using Parallel-Meta 3 (71), a software package for comprehensive taxonomical and functional comparison of microbial communities. Clustering of OTUs was conducted at the 97% similarity level using a preclustered version of the GreenGenes database (72). To perform taxonomic classifications at the species level for staphylococcal species, staphylococcal sequences were determined to the species level by alignment to a curated collection of staphylococcal reference sequences from complete genome sequences and type strains. Finally, each sequence was assigned a taxonomic label at the species level (such as S. aureus, S. epidermidis, Staphylococcus capitis, and S. hominis) based on the consensus call of sequence alignments with the lowest edit distance between a query and reference. The α diversity was calculated by Shannon index and Chao1, and the distance between each pairs of skin microbiota was computed based on the weighted Meta-Storms algorithm (73). For a certain genus to be considered “present,” it has to be of at least 0.01% abundance in at least 50% of the hosts within a city. Those genera with <0.01% abundance were merged together and referred to as “other genera”; on the other hand, those genera with <50% prevalence among the hosts within a city were not considered further (74). As for β-diversity, Meta-Storms distance (which is integrated in PM3 [71]) was used to quantify the differences between any two samples. The Meta-Storms scoring function is a phylogeny-based algorithm that quantitatively evaluates the biological similarity/distance between the microbiome samples on the OTU level (73). In parallel with above efforts, the contribution of conditionally rate taxa (CRT) to discrimination of originated city or AD status was quantified using CRT detection scripts (v1.0) with default parameters (75).

The other statistical analysis, e.g., Kruskal-Wallis test, Wilcoxon rank sum test, and permutational multivariate analysis of variance (PERMANOVA), were performed via the R scripts integrated in PM3. The scripts take advantage of using standard functions of kruskal.test and wilcox.test, as well as the adonis function in the R package of vegan. The rarefaction analysis and Shannon diversity index were used to estimate the richness and diversity of species. The relative abundance of differential taxonomic groups were visualized by “pheatmap” in the “pheatmap” R package. Differences in the relative abundance of taxonomic groups at the genus level between samples were evaluated with Wilcoxon rank sum test. False-discovery rate (FDR) values were estimated using the Benjamini-Hochberg method to control for multiple testing. *P* values less than 0.05 were considered statistically significant.

### Building the diagnostic models of atopic dermatitis.

The N top-ranking AD-discriminatory taxa that led to reasonably good fit were identified based on “rfcv” function in the randomForest package (https://cran.r-project.org/web/packages/randomForest/index.html). Random Forests models were trained to identify disease status in the training set which included samples from the ‘‘healthy’’ and the ‘‘lesional’’ groups using the taxonomy profiles. The results were evaluated with a 10-fold cross-validation approach, and model performance was evaluated by ROC. Default parameters of the R implementation of algorithm were applied (ntree = 5,000, using default mtry of p/3, where p is the number of input taxa). To construct and optimize the MiSH, we tested how taxonomical levels influence the performance of RF model. Using the profiles of genus, the performance of models based on microbiota was further evaluated with a 10-fold cross-validation approach. In 10-fold cross-validation, the original samples were randomly partitioned into 10 equal-sized subsamples. Of the 10 subsamples, a single subsample is retained as the validation data for testing the model, and the remaining nine subsamples were used as training data. The cross-validation process was then repeated for 10 times, and the average of probability was reported as the result. Based on optimization that selects the taxonomy level that maximizes model performance, Random Forest models were trained to identify disease status using the taxonomy profiles on the genus level. A receiver operating characteristic (ROC) curve was then used to illustrate the diagnostic performance of RF model (https://cran.r-project.org/web/packages/pROC/index.html). In the ROC plots, *x* axis represents true-positive rate (TPR, or sensitivity), *y* axis stands for false-positive rate (FPR, or specificity), and area under the ROC curve (AUC) was calculated to summarize performance of the RF model.

### Data availability.

The sequence data in this study have been submitted to the Sequence Read Archive (https://www.ncbi.nlm.nih.gov/sra) and can be accessed through the BioProject numbers PRJNA445780 and PRJNA268694.
