# Supplementary material for: A Microbiome-Based Index for Assessing Skin Health and Treatment Effects for Atopic Dermatitis in Children
Source: mSystems. 2019 Aug 20;4(4):e00293-19. doi: 10.1128/mSystems.00293-19 (PMC6702293; doi:10.1128/mSystems.00293-19)
Supplement: TABLE S1 [file mSystems.00293-19-st001.docx]

Supplementary Table1 for

**A Microbiome-based Index for Assessing Skin Health and Treatment effect for Atopic Dermatitis in Children**

Table S1. Details of participants and samples from the three cities of Beijing, Qingdao and Denver

**Table S1. Details of participants and samples from the three cities of Beijing, Qingdao and Denver.**

| SampleID | Status | Gender | City | Site | Age | SCORAD |
| --- | --- | --- | --- | --- | --- | --- |
| S_1H | Healthy | Boy | Qingdao | Shank | 3.0 | 0.00 |
| S_10H | Healthy | Girl | Qingdao | Arm | 3.5 | 0.00 |
| S_13H | Healthy | Boy | Qingdao | Arm | 4.0 | 0.00 |
| S_14H | Healthy | Boy | Qingdao | Shank | 3.5 | 0.00 |
| S_15H | Healthy | Boy | Qingdao | Shank | 3.0 | 0.00 |
| S_16H | Healthy | Girl | Qingdao | Arm | 3.0 | 0.00 |
| S_17H | Healthy | Girl | Qingdao | Arm | 3.5 | 0.00 |
| S_18H | Healthy | Girl | Qingdao | Shank | 3.0 | 0.00 |
| S_19H | Healthy | Girl | Qingdao | Arm | 4.0 | 0.00 |
| S_20H | Healthy | Girl | Qingdao | Arm | 3.0 | 0.00 |
| S_21H | Healthy | Girl | Qingdao | Arm | 3.5 | 0.00 |
| S_22H | Healthy | Girl | Qingdao | Arm | 3.0 | 0.00 |
| S_25H | Healthy | Girl | Qingdao | Arm | 3.0 | 0.00 |
| S_26H | Healthy | Boy | Qingdao | Arm | 4.0 | 0.00 |
| S_27H | Healthy | Girl | Qingdao | Arm | 3.0 | 0.00 |
| S_31H | Healthy | Boy | Qingdao | Arm | 3.5 | 0.00 |
| S_32H | Healthy | Boy | Qingdao | Arm | 3.0 | 0.00 |
| S_33H | Healthy | Boy | Qingdao | Arm | 3.0 | 0.00 |
| S_34H | Healthy | Girl | Qingdao | Arm | 3.5 | 0.00 |
| S_35H | Healthy | Girl | Qingdao | Shank | 4.0 | 0.00 |
| S_36H | Healthy | Girl | Qingdao | Arm | 4.0 | 0.00 |
| S_37H | Healthy | Boy | Qingdao | Arm | 3.0 | 0.00 |
| S_38H | Healthy | Boy | Qingdao | Arm | 3.0 | 0.00 |
| S_39H | Healthy | Girl | Qingdao | Arm | 3.0 | 0.00 |
| S_4H | Healthy | Girl | Qingdao | Arm | 3.5 | 0.00 |
| S_40H | Healthy | Girl | Qingdao | Arm | 3.0 | 0.00 |
| S_41H | Healthy | Girl | Qingdao | Arm | 3.0 | 0.00 |
| S_42H | Healthy | Girl | Qingdao | Arm | 3.5 | 0.00 |
| S_43H | Healthy | Girl | Qingdao | Arm | 3.5 | 0.00 |
| S_44H | Healthy | Girl | Qingdao | Arm | 3.0 | 0.00 |
| S_10_1 | Lesional | Boy | Qingdao | Arm | 2.0 | 32.75 |
| S_14_1 | Lesional | Girl | Qingdao | Arm | 4.5 | 26.10 |
| S_15_3 | Lesional | Boy | Qingdao | Shank | 4.0 | 10.90 |
| S_17_4 | Lesional | Boy | Qingdao | Shank | 11.0 | 39.40 |
| S_2_1 | Lesional | Boy | Qingdao | Arm | 7.0 | 25.50 |
| S_20_1 | Lesional | Girl | Qingdao | Arm | 6.0 | 26.50 |
| S_21_2 | Lesional | Girl | Qingdao | Arm | 4.0 | 43.20 |
| S_22_4 | Lesional | Girl | Qingdao | Shank | 3.0 | 22.20 |
| S_23_1 | Lesional | Boy | Qingdao | Arm | 2.0 | 24.55 |
| S_27_1 | Lesional | Boy | Qingdao | Arm | 7.0 | 20.90 |
| S_29_1 | Lesional | Girl | Qingdao | Arm | 6.0 | 25.60 |
| S_3_1 | Lesional | Girl | Qingdao | Arm | 2.0 | 15.25 |
| S_32_2 | Lesional | Girl | Qingdao | Arm | 4.0 | 24.60 |
| S_34_1 | Lesional | Girl | Qingdao | Arm | 14.0 | 24.60 |
| S_35_1 | Lesional | Boy | Qingdao | Arm | 9.0 | 38.50 |
| S_36_1 | Lesional | Girl | Qingdao | Arm | 5.0 | 14.60 |
| S_37_2 | Lesional | Girl | Qingdao | Arm | 0.5 | 22.80 |
| S_38_1 | Lesional | Boy | Qingdao | Shank | 14.0 | 27.70 |
| S_4_1 | Lesional | Boy | Qingdao | Arm | 6.0 | 19.10 |
| S_42_3 | Lesional | Girl | Qingdao | Shank | 4.0 | 25.90 |
| S_43_3 | Lesional | Girl | Qingdao | Shank | 2.0 | 18.80 |
| S_45_3 | Lesional | Girl | Qingdao | Shank | 2.5 | 33.30 |
| S_47_1 | Lesional | Boy | Qingdao | Arm | 8.0 | 29.70 |
| S_48_3 | Lesional | Girl | Qingdao | Shank | 2.0 | 39.10 |
| S_49_1 | Lesional | Boy | Qingdao | Arm | 5.0 | 28.80 |
| S_50_3 | Lesional | Girl | Qingdao | Shank | 4.0 | 21.85 |
| S_53_3 | Lesional | Boy | Qingdao | Shank | 3.0 | 46.60 |
| SRR4384152 | Healthy | NA | Denver | Arm | 3~12 | 0.00 |
| SRR4384153 | Healthy | NA | Denver | Arm | 3~12 | 0.00 |
| SRR4384154 | Healthy | NA | Denver | Arm | 3~12 | 0.00 |
| SRR4384155 | Healthy | NA | Denver | Arm | 3~12 | 0.00 |
| SRR4384157 | Healthy | NA | Denver | Arm | 3~12 | 0.00 |
| SRR4384158 | Healthy | NA | Denver | Arm | 3~12 | 0.00 |
| SRR4384347 | Healthy | NA | Denver | Arm | 3~12 | 0.00 |
| SRR4384355 | Healthy | NA | Denver | Arm | 3~12 | 0.00 |
| SRR4384358 | Healthy | NA | Denver | Arm | 3~12 | 0.00 |
| SRR4384399 | Healthy | NA | Denver | Arm | 3~12 | 0.00 |
| SRR4384400 | Healthy | NA | Denver | Arm | 3~12 | 0.00 |
| SRR4384401 | Healthy | NA | Denver | Arm | 3~12 | 0.00 |
| SRR4384403 | Healthy | NA | Denver | Arm | 3~12 | 0.00 |
| SRR4384102 | Lesional | NA | Denver | Arm | 3~12 | NA |
| SRR4384104 | Lesional | NA | Denver | Arm | 3~12 | NA |
| SRR4384115 | Lesional | NA | Denver | Arm | 3~12 | NA |
| SRR4384124 | Lesional | NA | Denver | Arm | 3~12 | NA |
| SRR4384137 | Lesional | NA | Denver | Arm | 3~12 | NA |
| SRR4384139 | Lesional | NA | Denver | Arm | 3~12 | NA |
| SRR4384143 | Lesional | NA | Denver | Arm | 3~12 | NA |
| SRR4384146 | Lesional | NA | Denver | Arm | 3~12 | NA |
| SRR4384148 | Lesional | NA | Denver | Arm | 3~12 | NA |
| SRR4384162 | Lesional | NA | Denver | Arm | 3~12 | NA |
| SRR4384164 | Lesional | NA | Denver | Arm | 3~12 | NA |
| SRR4384171 | Lesional | NA | Denver | Arm | 3~12 | NA |
| SRR4384176 | Lesional | NA | Denver | Arm | 3~12 | NA |
| SRR4384178 | Lesional | NA | Denver | Arm | 3~12 | NA |
| SRR4384185 | Lesional | NA | Denver | Arm | 3~12 | NA |
| SRR4384196 | Lesional | NA | Denver | Arm | 3~12 | NA |
| SRR4384198 | Lesional | NA | Denver | Arm | 3~12 | NA |
| SRR4384212 | Lesional | NA | Denver | Arm | 3~12 | NA |
| SRR4384214 | Lesional | NA | Denver | Arm | 3~12 | NA |
| SRR4384215 | Lesional | NA | Denver | Arm | 3~12 | NA |
| SRR4384225 | Lesional | NA | Denver | Arm | 3~12 | NA |
| SRR4384242 | Lesional | NA | Denver | Arm | 3~12 | NA |
| SRR4384244 | Lesional | NA | Denver | Arm | 3~12 | NA |
| SRR4384256 | Lesional | NA | Denver | Arm | 3~12 | NA |
| SRR4384258 | Lesional | NA | Denver | Arm | 3~12 | NA |
| SRR4384261 | Lesional | NA | Denver | Arm | 3~12 | NA |
| SRR4384263 | Lesional | NA | Denver | Arm | 3~12 | NA |
| SRR4384265 | Lesional | NA | Denver | Arm | 3~12 | NA |
| SRR4384267 | Lesional | NA | Denver | Arm | 3~12 | NA |
| SRR4384269 | Lesional | NA | Denver | Arm | 3~12 | NA |
| SRR4384283 | Lesional | NA | Denver | Arm | 3~12 | NA |
| SRR4384287 | Lesional | NA | Denver | Arm | 3~12 | NA |
| SRR4384292 | Lesional | NA | Denver | Arm | 3~12 | NA |
| SRR4384311 | Lesional | NA | Denver | Arm | 3~12 | NA |
| SRR4384313 | Lesional | NA | Denver | Arm | 3~12 | NA |
| SRR4384320 | Lesional | NA | Denver | Arm | 3~12 | NA |
| SRR4384330 | Lesional | NA | Denver | Arm | 3~12 | NA |
| SRR4384332 | Lesional | NA | Denver | Arm | 3~12 | NA |
| SRR4384334 | Lesional | NA | Denver | Arm | 3~12 | NA |
| SRR4384337 | Lesional | NA | Denver | Arm | 3~12 | NA |
| SRR4384362 | Lesional | NA | Denver | Arm | 3~12 | NA |
| SRR4384364 | Lesional | NA | Denver | Arm | 3~12 | NA |
| SRR4384366 | Lesional | NA | Denver | Arm | 3~12 | NA |
| SRR4384370 | Lesional | NA | Denver | Arm | 3~12 | NA |
| SRR4384377 | Lesional | NA | Denver | Arm | 3~12 | NA |
| SRR4384384 | Lesional | NA | Denver | Arm | 3~12 | NA |
| SRR4384387 | Lesional | NA | Denver | Arm | 3~12 | NA |
| SRR4384389 | Lesional | NA | Denver | Arm | 3~12 | NA |
| SRR4384391 | Lesional | NA | Denver | Arm | 3~12 | NA |
| SRR4384405 | Lesional | NA | Denver | Arm | 3~12 | NA |
| SRR4384407 | Lesional | NA | Denver | Arm | 3~12 | NA |
| SRR4384409 | Lesional | NA | Denver | Arm | 3~12 | NA |
| SRR4384411 | Lesional | NA | Denver | Arm | 3~12 | NA |
| SRR4384413 | Lesional | NA | Denver | Arm | 3~12 | NA |
| SRR4384416 | Lesional | NA | Denver | Arm | 3~12 | NA |
| SRR4384418 | Lesional | NA | Denver | Arm | 3~12 | NA |
| SRR4384420 | Lesional | NA | Denver | Arm | 3~12 | NA |
| SRR4384422 | Lesional | NA | Denver | Arm | 3~12 | NA |
| SRR4384424 | Lesional | NA | Denver | Arm | 3~12 | NA |
| SRR4384103 | non_Lesional | NA | Denver | Arm | 3~12 | NA |
| SRR4384105 | non_Lesional | NA | Denver | Arm | 3~12 | NA |
| SRR4384116 | non_Lesional | NA | Denver | Arm | 3~12 | NA |
| SRR4384125 | non_Lesional | NA | Denver | Arm | 3~12 | NA |
| SRR4384138 | non_Lesional | NA | Denver | Arm | 3~12 | NA |
| SRR4384140 | non_Lesional | NA | Denver | Arm | 3~12 | NA |
| SRR4384144 | non_Lesional | NA | Denver | Arm | 3~12 | NA |
| SRR4384147 | non_Lesional | NA | Denver | Arm | 3~12 | NA |
| SRR4384149 | non_Lesional | NA | Denver | Arm | 3~12 | NA |
| SRR4384163 | non_Lesional | NA | Denver | Arm | 3~12 | NA |
| SRR4384165 | non_Lesional | NA | Denver | Arm | 3~12 | NA |
| SRR4384172 | non_Lesional | NA | Denver | Arm | 3~12 | NA |
| SRR4384177 | non_Lesional | NA | Denver | Arm | 3~12 | NA |
| SRR4384180 | non_Lesional | NA | Denver | Arm | 3~12 | NA |
| SRR4384186 | non_Lesional | NA | Denver | Arm | 3~12 | NA |
| SRR4384197 | non_Lesional | NA | Denver | Arm | 3~12 | NA |
| SRR4384199 | non_Lesional | NA | Denver | Arm | 3~12 | NA |
| SRR4384213 | non_Lesional | NA | Denver | Arm | 3~12 | NA |
| SRR4384226 | non_Lesional | NA | Denver | Arm | 3~12 | NA |
| SRR4384227 | non_Lesional | NA | Denver | Arm | 3~12 | NA |
| SRR4384243 | non_Lesional | NA | Denver | Arm | 3~12 | NA |
| SRR4384245 | non_Lesional | NA | Denver | Arm | 3~12 | NA |
| SRR4384255 | non_Lesional | NA | Denver | Arm | 3~12 | NA |
| SRR4384257 | non_Lesional | NA | Denver | Arm | 3~12 | NA |
| SRR4384260 | non_Lesional | NA | Denver | Arm | 3~12 | NA |
| SRR4384262 | non_Lesional | NA | Denver | Arm | 3~12 | NA |
| SRR4384264 | non_Lesional | NA | Denver | Arm | 3~12 | NA |
| SRR4384266 | non_Lesional | NA | Denver | Arm | 3~12 | NA |
| SRR4384268 | non_Lesional | NA | Denver | Arm | 3~12 | NA |
| SRR4384271 | non_Lesional | NA | Denver | Arm | 3~12 | NA |
| SRR4384281 | non_Lesional | NA | Denver | Arm | 3~12 | NA |
| SRR4384284 | non_Lesional | NA | Denver | Arm | 3~12 | NA |
| SRR4384312 | non_Lesional | NA | Denver | Arm | 3~12 | NA |
| SRR4384315 | non_Lesional | NA | Denver | Arm | 3~12 | NA |
| SRR4384321 | non_Lesional | NA | Denver | Arm | 3~12 | NA |
| SRR4384325 | non_Lesional | NA | Denver | Arm | 3~12 | NA |
| SRR4384326 | non_Lesional | NA | Denver | Arm | 3~12 | NA |
| SRR4384331 | non_Lesional | NA | Denver | Arm | 3~12 | NA |
| SRR4384333 | non_Lesional | NA | Denver | Arm | 3~12 | NA |
| SRR4384335 | non_Lesional | NA | Denver | Arm | 3~12 | NA |
| SRR4384361 | non_Lesional | NA | Denver | Arm | 3~12 | NA |
| SRR4384363 | non_Lesional | NA | Denver | Arm | 3~12 | NA |
| SRR4384365 | non_Lesional | NA | Denver | Arm | 3~12 | NA |
| SRR4384369 | non_Lesional | NA | Denver | Arm | 3~12 | NA |
| SRR4384376 | non_Lesional | NA | Denver | Arm | 3~12 | NA |
| SRR4384383 | non_Lesional | NA | Denver | Arm | 3~12 | NA |
| SRR4384386 | non_Lesional | NA | Denver | Arm | 3~12 | NA |
| SRR4384388 | non_Lesional | NA | Denver | Arm | 3~12 | NA |
| SRR4384390 | non_Lesional | NA | Denver | Arm | 3~12 | NA |
| SRR4384406 | non_Lesional | NA | Denver | Arm | 3~12 | NA |
| SRR4384408 | non_Lesional | NA | Denver | Arm | 3~12 | NA |
| SRR4384410 | non_Lesional | NA | Denver | Arm | 3~12 | NA |
| SRR4384412 | non_Lesional | NA | Denver | Arm | 3~12 | NA |
| SRR4384414 | non_Lesional | NA | Denver | Arm | 3~12 | NA |
| SRR4384419 | non_Lesional | NA | Denver | Arm | 3~12 | NA |
| SRR4384421 | non_Lesional | NA | Denver | Arm | 3~12 | NA |
| SRR4384423 | non_Lesional | NA | Denver | Arm | 3~12 | NA |
| SRR4384425 | non_Lesional | NA | Denver | Arm | 3~12 | NA |
| S_14_2 | non_Lesional | Girl | Qingdao | Arm | 4.5 | 26.10 |
| S_14_3 | non_Lesional | Girl | Qingdao | Shank | 4.5 | 26.10 |
| S_14_4 | non_Lesional | Girl | Qingdao | Shank | 4.5 | 26.10 |
| S_17_3 | non_Lesional | Boy | Qingdao | Shank | 11.0 | 39.40 |
| S_2_2 | non_Lesional | Boy | Qingdao | Arm | 7.0 | 25.50 |
| S_2_3 | non_Lesional | Boy | Qingdao | Shank | 7.0 | 25.50 |
| S_20_2 | non_Lesional | Girl | Qingdao | Arm | 6.0 | 26.50 |
| S_21_3 | non_Lesional | Girl | Qingdao | Shank | 4.0 | 43.20 |
| S_21_4 | non_Lesional | Girl | Qingdao | Shank | 4.0 | 43.20 |
| S_22_1 | non_Lesional | Girl | Qingdao | Arm | 3.0 | 22.20 |
| S_22_2 | non_Lesional | Girl | Qingdao | Arm | 3.0 | 22.20 |
| S_23_2 | non_Lesional | Boy | Qingdao | Arm | 2.0 | 24.55 |
| S_23_3 | non_Lesional | Boy | Qingdao | Shank | 2.0 | 24.55 |
| S_23_4 | non_Lesional | Boy | Qingdao | Shank | 2.0 | 24.55 |
| S_24_2 | non_Lesional | Boy | Qingdao | Arm | 8.0 | 29.15 |
| S_27_2 | non_Lesional | Boy | Qingdao | Arm | 7.0 | 20.90 |
| S_28_2 | non_Lesional | Girl | Qingdao | Arm | 4.0 | 31.20 |
| S_28_3 | non_Lesional | Girl | Qingdao | Shank | 4.0 | 31.20 |
| S_28_4 | non_Lesional | Girl | Qingdao | Shank | 4.0 | 31.20 |
| S_29_2 | non_Lesional | Girl | Qingdao | Arm | 6.0 | 25.60 |
| S_29_3 | non_Lesional | Girl | Qingdao | Shank | 6.0 | 25.60 |
| S_29_4 | non_Lesional | Girl | Qingdao | Shank | 6.0 | 25.60 |
| S_3_3 | non_Lesional | Girl | Qingdao | Shank | 2.0 | 15.25 |
| S_30_2 | non_Lesional | Boy | Qingdao | Arm | 5.0 | 25.60 |
| S_30_3 | non_Lesional | Boy | Qingdao | Shank | 5.0 | 25.60 |
| S_30_4 | non_Lesional | Boy | Qingdao | Shank | 5.0 | 25.60 |
| S_31_3 | non_Lesional | Girl | Qingdao | Shank | 4.0 | 14.45 |
| S_31_4 | non_Lesional | Girl | Qingdao | Shank | 4.0 | 14.45 |
| S_33_2 | non_Lesional | Girl | Qingdao | Arm | 6.0 | 24.60 |
| S_33_3 | non_Lesional | Girl | Qingdao | Shank | 6.0 | 24.60 |
| S_33_4 | non_Lesional | Girl | Qingdao | Shank | 6.0 | 24.60 |
| S_34_2 | non_Lesional | Girl | Qingdao | Arm | 12.0 | 24.60 |
| S_34_3 | non_Lesional | Girl | Qingdao | Shank | 12.0 | 24.60 |
| S_34_4 | non_Lesional | Girl | Qingdao | Shank | 12.0 | 24.60 |
| S_35_2 | non_Lesional | Boy | Qingdao | Arm | 9.0 | 38.50 |
| S_35_3 | non_Lesional | Boy | Qingdao | Shank | 9.0 | 38.50 |
| S_35_4 | non_Lesional | Boy | Qingdao | Shank | 9.0 | 38.50 |
| S_36_2 | non_Lesional | Girl | Qingdao | Arm | 5.0 | 14.60 |
| S_36_3 | non_Lesional | Girl | Qingdao | Shank | 5.0 | 14.60 |
| S_36_4 | non_Lesional | Girl | Qingdao | Shank | 5.0 | 14.60 |
| S_37_1 | non_Lesional | Girl | Qingdao | Arm | 0.5 | 22.80 |
| S_39_3 | non_Lesional | Girl | Qingdao | Arm | 4.0 | 27.70 |
| S_4_2 | non_Lesional | Boy | Qingdao | Arm | 6.0 | 19.10 |
| S_4_3 | non_Lesional | Boy | Qingdao | Shank | 6.0 | 19.10 |
| S_42_1 | non_Lesional | Girl | Qingdao | Arm | 4.0 | 25.90 |
| S_42_2 | non_Lesional | Girl | Qingdao | Arm | 4.0 | 25.90 |
| S_42_4 | non_Lesional | Girl | Qingdao | Shank | 4.0 | 25.90 |
| S_43_4 | non_Lesional | Girl | Qingdao | Shank | 2.0 | 18.80 |
| S_44_4 | non_Lesional | Girl | Qingdao | Shank | 4.0 | 28.20 |
| S_45_1 | non_Lesional | Girl | Qingdao | Arm | 2.5 | 33.30 |
| S_45_2 | non_Lesional | Girl | Qingdao | Arm | 2.5 | 33.30 |
| S_46_2 | non_Lesional | Girl | Qingdao | Arm | 4.0 | 32.20 |
| S_46_3 | non_Lesional | Girl | Qingdao | Shank | 4.0 | 32.20 |
| S_46_4 | non_Lesional | Girl | Qingdao | Shank | 4.0 | 32.20 |
| S_47_2 | non_Lesional | Boy | Qingdao | Arm | 8.0 | 29.70 |
| S_47_3 | non_Lesional | Boy | Qingdao | Shank | 8.0 | 29.70 |
| S_47_4 | non_Lesional | Boy | Qingdao | Shank | 8.0 | 29.70 |
| S_48_4 | non_Lesional | Girl | Qingdao | Shank | 2.0 | 39.10 |
| S_49_2 | non_Lesional | Boy | Qingdao | Arm | 5.0 | 28.80 |
| S_49_3 | non_Lesional | Boy | Qingdao | Shank | 5.0 | 28.80 |
| S_49_4 | non_Lesional | Boy | Qingdao | Shank | 5.0 | 28.80 |
| S_50_1 | non_Lesional | Girl | Qingdao | Arm | 4.0 | 21.85 |
| S_50_4 | non_Lesional | Girl | Qingdao | Shank | 4.0 | 21.85 |
| S_51_1 | non_Lesional | Boy | Qingdao | Arm | 2.0 | 45.00 |
| S_51_2 | non_Lesional | Boy | Qingdao | Arm | 2.0 | 45.00 |
| S_51_3 | non_Lesional | Boy | Qingdao | Shank | 2.0 | 45.00 |
| S_53_1 | non_Lesional | Boy | Qingdao | Arm | 3.0 | 30.70 |
| S_53_2 | non_Lesional | Boy | Qingdao | Arm | 3.0 | 30.70 |
| S_56_1 | non_Lesional | Girl | Qingdao | Arm | 4.0 | 17.20 |
| S_56_2 | non_Lesional | Girl | Qingdao | Arm | 4.0 | 17.20 |
| S_S1001N | non_Lesional | Girl | Beijing | Arm | 8.0 | 16.52 |
| S_S1002N | non_Lesional | Boy | Beijing | Arm | 8.0 | NA |
| S_S1005N | non_Lesional | Boy | Beijing | Popliteal Fossa | 4.0 | 25.29 |
| S_S1008N | non_Lesional | Girl | Beijing | Shank | 8.0 | 21.48 |
| S_S1020N | non_Lesional | Girl | Beijing | Elbow | 4.0 | 32.38 |
| S_S1024N | non_Lesional | Boy | Beijing | Shank | 4.0 | 47.24 |
| S_S1027N | non_Lesional | Girl | Beijing | Antecubital | 4.0 | 14.67 |
| S_S1030N | non_Lesional | Girl | Beijing | Arm | 5.0 | 44.14 |
| S_S1035N | non_Lesional | Girl | Beijing | Popliteal Fossa | 4.0 | NA |
| S_S1039N | non_Lesional | Girl | Beijing | Popliteal Fossa | 4.0 | 6.62 |
| S_S1050N | non_Lesional | Boy | Beijing | Knee | 6.0 | 33.50 |
| S_S1052N | non_Lesional | Boy | Beijing | Antecubital | 5.0 | 22.82 |
| S_S1053N | non_Lesional | Boy | Beijing | Neck | 8.0 | 2.85 |
| S_S1056N | non_Lesional | Boy | Beijing | Arm | 4.0 | 41.48 |
| S_S1060N | non_Lesional | Girl | Beijing | Knee | 4.0 | 20.62 |
| S_S1063N | non_Lesional | Boy | Beijing | Shank | 4.0 | 36.55 |
| S_S1064N | non_Lesional | Girl | Beijing | Popliteal Fossa | 4.0 | 7.79 |
| S_S1069N | non_Lesional | Girl | Beijing | Antecubital | 6.0 | 36.50 |
| S_S1070N | non_Lesional | Boy | Beijing | Antecubital | 5.0 | 1.95 |
| S_S1072N | non_Lesional | Girl | Beijing | Popliteal Fossa | 5.0 | 10.65 |
| S_S1076N | non_Lesional | Boy | Beijing | Shank | 4.0 | 6.76 |
| S_S1077N | non_Lesional | Boy | Beijing | Shank | 6.0 | 19.94 |
| S_S1078N | non_Lesional | Boy | Beijing | Knee | 8.0 | 13.31 |
| S_S1080N | non_Lesional | Boy | Beijing | Antecubital | 10.0 | 10.35 |
| S_S1084N | non_Lesional | Boy | Beijing | Arm | 5.0 | 9.80 |
| S_S1087N | non_Lesional | Boy | Beijing | Shank | 7.0 | 15.16 |
| S_S1089N | non_Lesional | Boy | Beijing | Shank | 11.0 | 20.31 |
| S_S1098H | Healthy | Girl | Beijing | Shank | 8.0 | 0.00 |
| S_S1099H | Healthy | Girl | Beijing | Shank | 8.0 | 0.00 |
| S_S1100H | Healthy | Girl | Beijing | Antecubital | 6.0 | 0.00 |
| S_S1101H | Healthy | Boy | Beijing | Arm | 8.0 | 0.00 |
| S_S1102H | Healthy | Boy | Beijing | Shank | 4.0 | 0.00 |
| S_S1103H | Healthy | Boy | Beijing | Arm | 4.0 | 0.00 |
| S_S1104H | Healthy | Boy | Beijing | Antecubital | 5.0 | 0.00 |
| S_S1105H | Healthy | Boy | Beijing | Knee | 6.0 | 0.00 |
| S_S1106H | Healthy | Boy | Beijing | Neck | 8.0 | 0.00 |
| S_S1107H | Healthy | Boy | Beijing | Shank | 8.0 | 0.00 |
| S_S1108H | Healthy | Boy | Beijing | Shank | 7.0 | 0.00 |
| S_S1109H | Healthy | Boy | Beijing | Shank | 6.0 | 0.00 |
| S_S1110H | Healthy | Girl | Beijing | Antecubital | 6.0 | 0.00 |
| S_S1111H | Healthy | Girl | Beijing | Arm | 8.0 | 0.00 |
| S_S1113H | Healthy | Girl | Beijing | Popliteal Fossa | 4.0 | 0.00 |
| S_S1114H | Healthy | Boy | Beijing | Shank | 5.0 | 0.00 |
| S_S1115H | Healthy | Boy | Beijing | Popliteal Fossa | 4.0 | 0.00 |
| S_S1116H | Healthy | Boy | Beijing | Arm | 5.0 | 0.00 |
| S_S1117H | Healthy | Boy | Beijing | Antecubital | 5.0 | 0.00 |
| S_S1119H | Healthy | Boy | Beijing | Shank | 4.0 | 0.00 |
| S_S1121H | Healthy | Girl | Beijing | Popliteal Fossa | 4.0 | 0.00 |
| S_S1124H | Healthy | Girl | Beijing | Arm | 5.0 | 0.00 |
| S_S1126H | Healthy | Girl | Beijing | Popliteal Fossa | 5.0 | 0.00 |
| S_S1128H | Healthy | Girl | Beijing | Knee | 4.0 | 0.00 |
| S_S1130H | Healthy | Girl | Beijing | Arm | 6.0 | 0.00 |
| S_S1131H | Healthy | Girl | Beijing | Arm | 6.0 | 0.00 |
| S_S1132H | Healthy | Boy | Beijing | Arm | 6.0 | 0.00 |
| S1001N | non_Lesional | Girl | Beijing | Arm | 8.0 | 41.83 |
| S1005N | non_Lesional | Boy | Beijing | Popliteal Fossa | 4.0 | 65.75 |
| S1020N | non_Lesional | Girl | Beijing | Elbow | 4.0 | 41.23 |
| S1030N | non_Lesional | Girl | Beijing | Arm | 5.0 | 46.39 |
| S1036N | non_Lesional | Boy | Beijing | Arm | 8.0 | 0.00 |
| S1039N | non_Lesional | Girl | Beijing | Popliteal Fossa | 4.0 | 33.40 |
| S1050N | non_Lesional | Boy | Beijing | Knee | 6.0 | 73.00 |
| S1053N | non_Lesional | Boy | Beijing | Neck | 8.0 | 13.85 |
| S1056N | non_Lesional | Boy | Beijing | Arm | 4.0 | 48.81 |
| S1060N | non_Lesional | Girl | Beijing | Knee | 4.0 | 44.82 |
| S1061N | non_Lesional | Boy | Beijing | Shank | 11.0 | 20.55 |
| S1063N | non_Lesional | Boy | Beijing | Shank | 4.0 | 70.12 |
| S1064N | non_Lesional | Girl | Beijing | Popliteal Fossa | 4.0 | 38.55 |
| S1069N | non_Lesional | Girl | Beijing | Antecubital | 6.0 | 55.80 |
| S1072N | non_Lesional | Girl | Beijing | Popliteal Fossa | 5.0 | 33.57 |
| S1078N | non_Lesional | Boy | Beijing | Knee | 8.0 | 42.24 |
| S1080N | non_Lesional | Boy | Beijing | Antecubital | 10.0 | 28.30 |
| S1084N | non_Lesional | Boy | Beijing | Arm | 5.0 | 28.30 |
| S1087N | non_Lesional | Boy | Beijing | Shank | 7.0 | NA |
| S1089N | non_Lesional | Boy | Beijing | Shank | 11.0 | 28.88 |
| S_S1001L | Lesional | Girl | Beijing | Arm | 8.0 | 16.52 |
| S_S1005L | Lesional | Boy | Beijing | Popliteal Fossa | 4.0 | 25.29 |
| S_S1008L | Lesional | Girl | Beijing | Shank | 8.0 | 21.48 |
| S_S1020L | Lesional | Girl | Beijing | Elbow | 4.0 | 32.38 |
| S_S1024L | Lesional | Boy | Beijing | Shank | 4.0 | 47.24 |
| S_S1027L | Lesional | Girl | Beijing | Antecubital | 4.0 | 14.67 |
| S_S1030L | Lesional | Girl | Beijing | Arm | 5.0 | 44.14 |
| S_S1050L | Lesional | Boy | Beijing | Knee | 6.0 | 33.50 |
| S_S1052L | Lesional | Boy | Beijing | Antecubital | 5.0 | 22.82 |
| S_S1053L | Lesional | Boy | Beijing | Neck | 8.0 | 2.85 |
| S_S1056L | Lesional | Boy | Beijing | Arm | 4.0 | 41.48 |
| S_S1058L | Lesional | Boy | Beijing | Shank | 6.0 | 36.65 |
| S_S1060L | Lesional | Girl | Beijing | Knee | 4.0 | 20.62 |
| S_S1063L | Lesional | Boy | Beijing | Shank | 4.0 | 36.55 |
| S_S1064L | Lesional | Girl | Beijing | Popliteal Fossa | 4.0 | 7.79 |
| S_S1069L | Lesional | Girl | Beijing | Antecubital | 6.0 | 36.50 |
| S_S1070L | Lesional | Boy | Beijing | Antecubital | 5.0 | 1.95 |
| S_S1072L | Lesional | Girl | Beijing | Popliteal Fossa | 5.0 | 10.65 |
| S_S1076L | Lesional | Boy | Beijing | Shank | 4.0 | 6.76 |
| S_S1077L | Lesional | Boy | Beijing | Shank | 6.0 | 19.94 |
| S_S1078L | Lesional | Boy | Beijing | Knee | 8.0 | 13.31 |
| S_S1084L | Lesional | Boy | Beijing | Arm | 5.0 | 9.80 |
| S_S1087L | Lesional | Boy | Beijing | Shank | 7.0 | 15.16 |
| S_S1089L | Lesional | Boy | Beijing | Shank | 11.0 | 20.31 |
| S1001L | Lesional | Girl | Beijing | Arm | 8.0 | 41.83 |
| S1002L | Lesional | Boy | Beijing | Arm | 8.0 | 42.40 |
| S1005L | Lesional | Boy | Beijing | Popliteal Fossa | 4.0 | 65.75 |
| S1020L | Lesional | Girl | Beijing | Elbow | 4.0 | 41.23 |
| S1024L | Lesional | Boy | Beijing | Shank | 4.0 | 42.91 |
| S1026L | Lesional | Girl | Beijing | Axilla | 6.0 | 0.00 |
| S1039L | Lesional | Girl | Beijing | Popliteal Fossa | 4.0 | 33.40 |
| S1049L | Lesional | Girl | Beijing | Arm | 6.0 | 51.80 |
| S1050L | Lesional | Boy | Beijing | Knee | 6.0 | 73.00 |
| S1052L | Lesional | Boy | Beijing | Antecubital | 5.0 | 35.12 |
| S1053L | Lesional | Boy | Beijing | Neck | 8.0 | 13.85 |
| S1056L | Lesional | Boy | Beijing | Arm | 4.0 | 48.81 |
| S1058L | Lesional | Boy | Beijing | Shank | 6.0 | 28.52 |
| S1063L | Lesional | Boy | Beijing | Shank | 4.0 | 70.12 |
| S1064L | Lesional | Girl | Beijing | Popliteal Fossa | 4.0 | 38.55 |
| S1069L | Lesional | Girl | Beijing | Antecubital | 6.0 | 55.80 |
| S1070L | Lesional | Boy | Beijing | Antecubital | 5.0 | 13.20 |
| S1072L | Lesional | Girl | Beijing | Popliteal Fossa | 5.0 | 33.57 |
| S1077L | Lesional | Boy | Beijing | Shank | 6.0 | 31.20 |
| S1078L | Lesional | Boy | Beijing | Knee | 8.0 | 42.24 |
| S1080L | Lesional | Boy | Beijing | Antecubital | 10.0 | 28.30 |
| S1084L | Lesional | Boy | Beijing | Arm | 5.0 | 28.30 |
| S1027L | Lesional | Girl | Beijing | Antecubital | 4.0 | 17.31 |
| S1030L | Lesional | Girl | Beijing | Arm | 5.0 | 46.39 |
| S1032L | Lesional | Girl | Beijing | Arm | 4.0 | 0.00 |
| S1036L | Lesional | Boy | Beijing | Arm | 8.0 | 0.00 |
| S1046L | Lesional | Boy | Beijing | Shank | 5.0 | 0.00 |
| S1060L | Lesional | Girl | Beijing | Knee | 4.0 | 44.82 |
| S1061L | Lesional | Boy | Beijing | Shank | 11.0 | 20.55 |
| S1089L | Lesional | Boy | Beijing | Shank | 11.0 | 28.88 |
