# Supplementary figures and images for: A Microbiome-Based Index for Assessing Skin Health and Treatment Effects for Atopic Dermatitis in Children
Source: mSystems. 2019 Aug 20;4(4):e00293-19. doi: 10.1128/mSystems.00293-19 (PMC6702293; doi:10.1128/mSystems.00293-19)

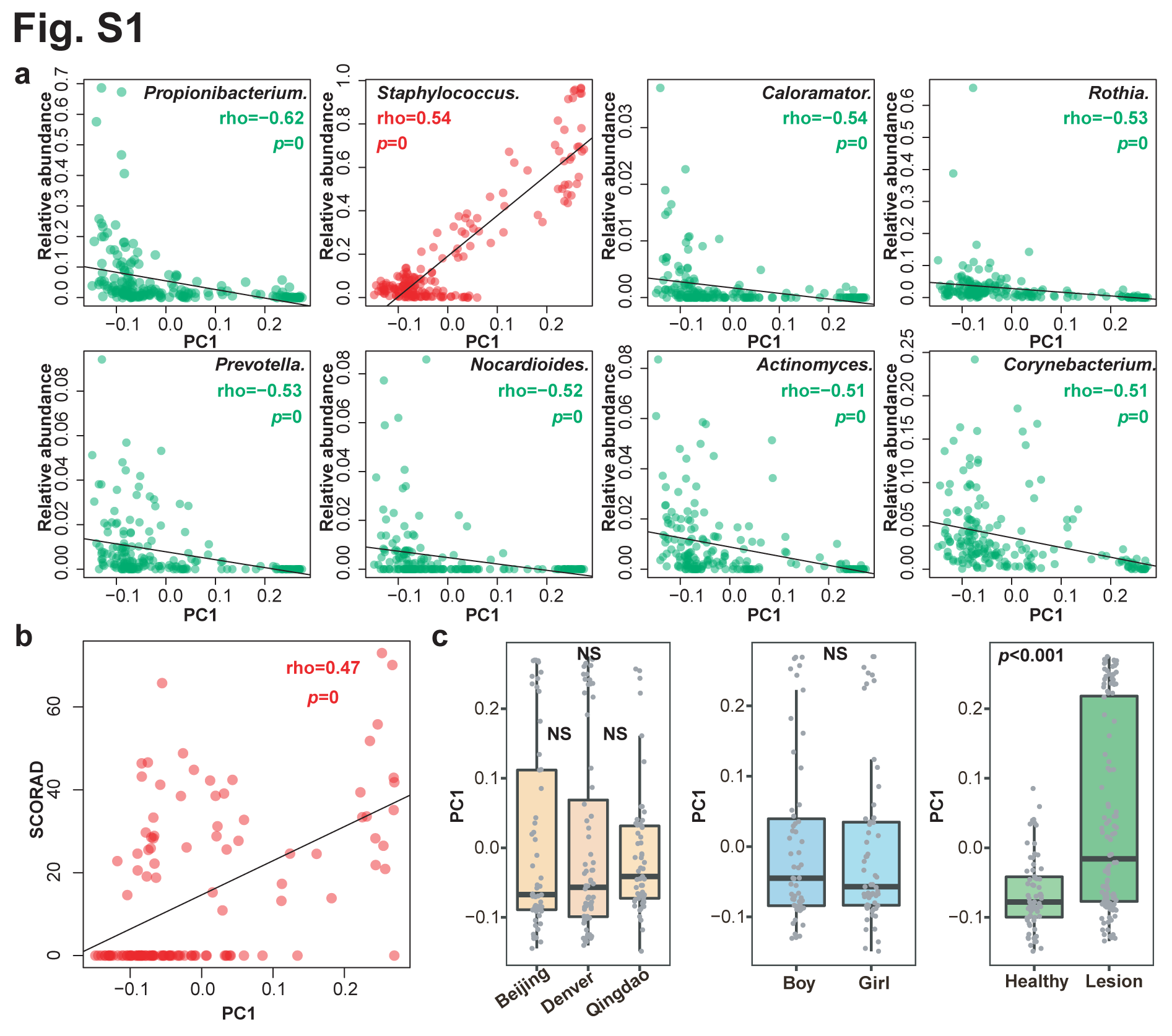

Supplement: FIG S1 [file mSystems.00293-19-sf001.tif]

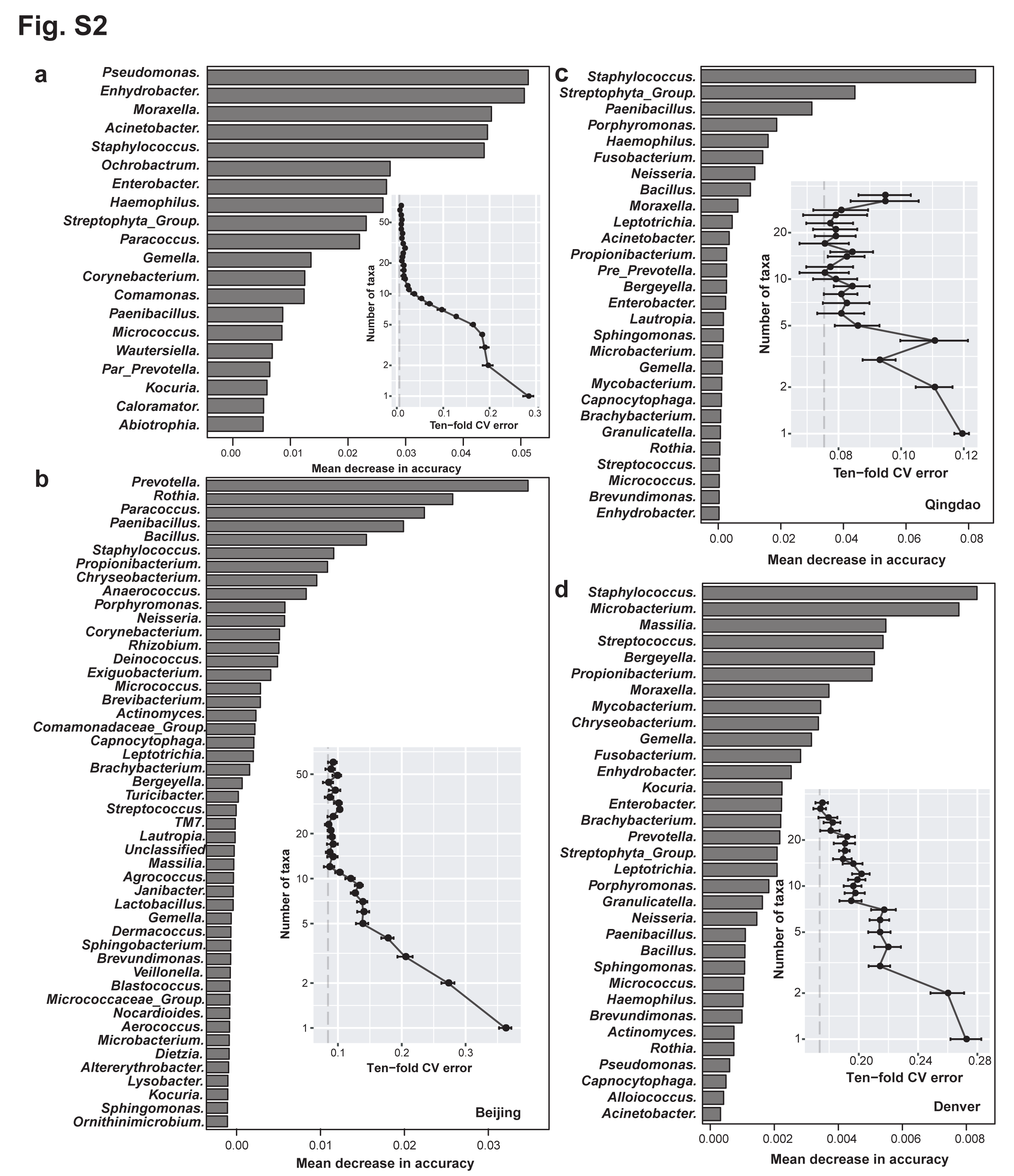

Supplement: FIG S2 [file mSystems.00293-19-sf002.tif]

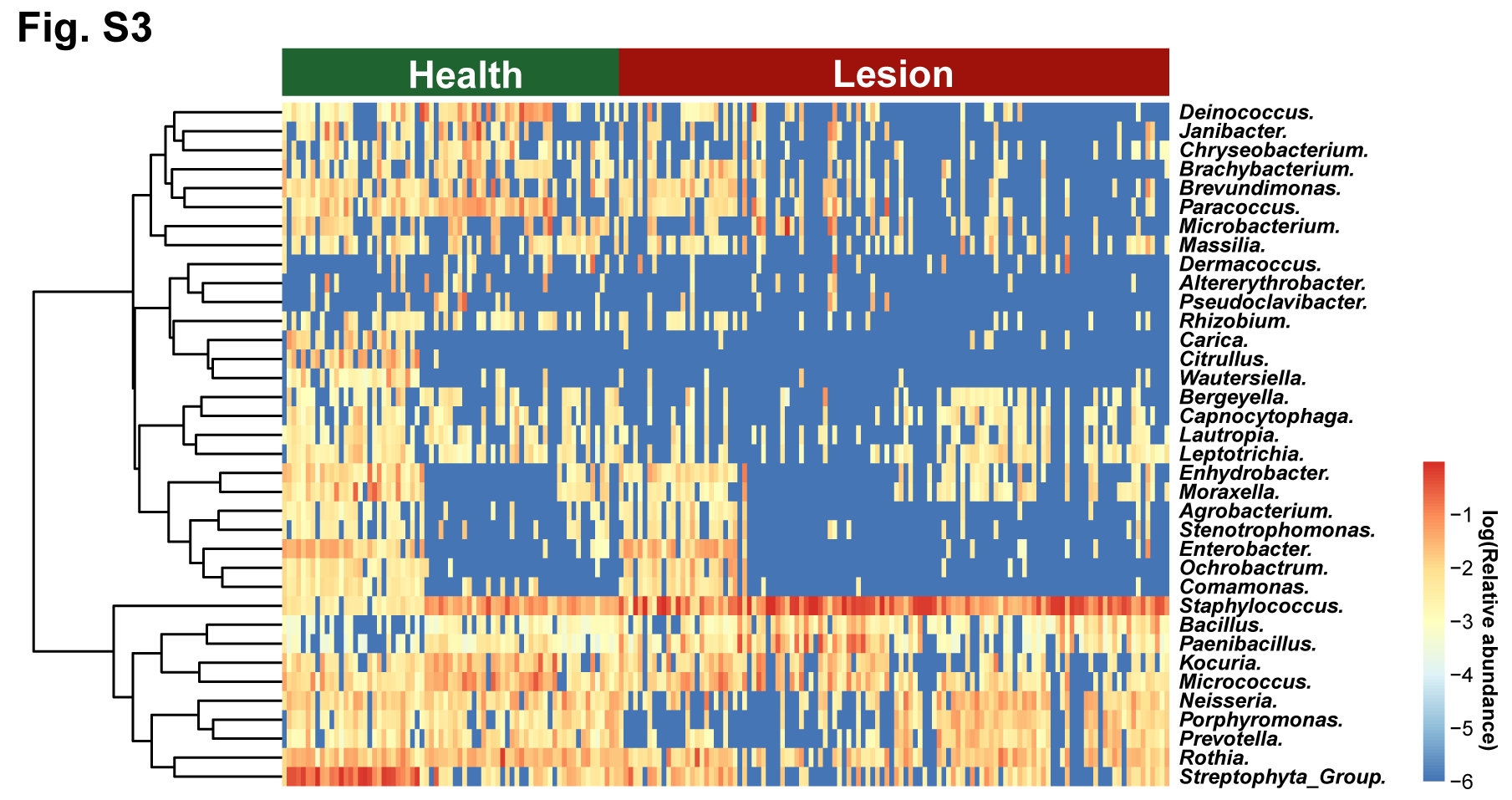

Supplement: FIG S3 [file mSystems.00293-19-sf003.tif]

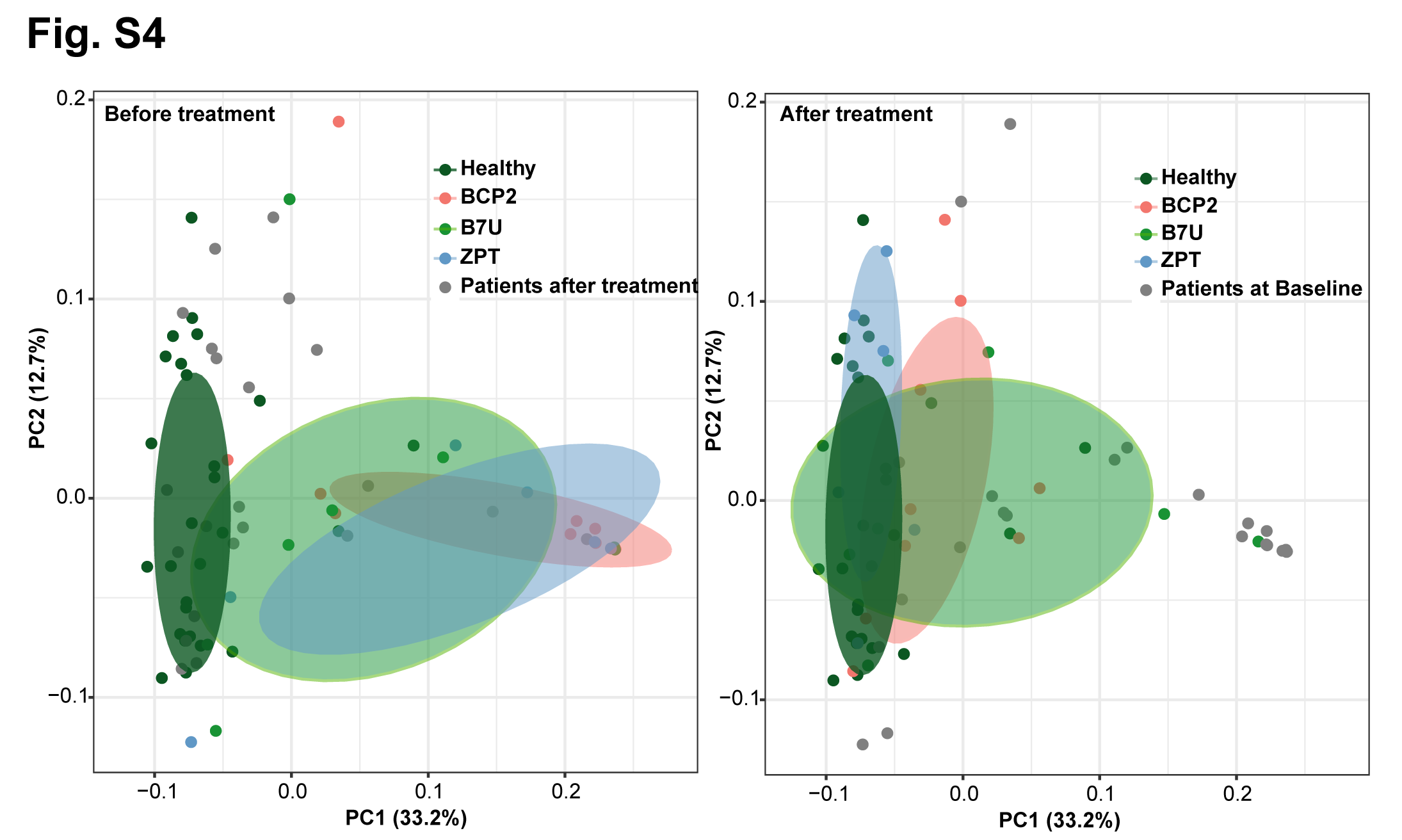

Supplement: FIG S4 [file mSystems.00293-19-sf004.tif]

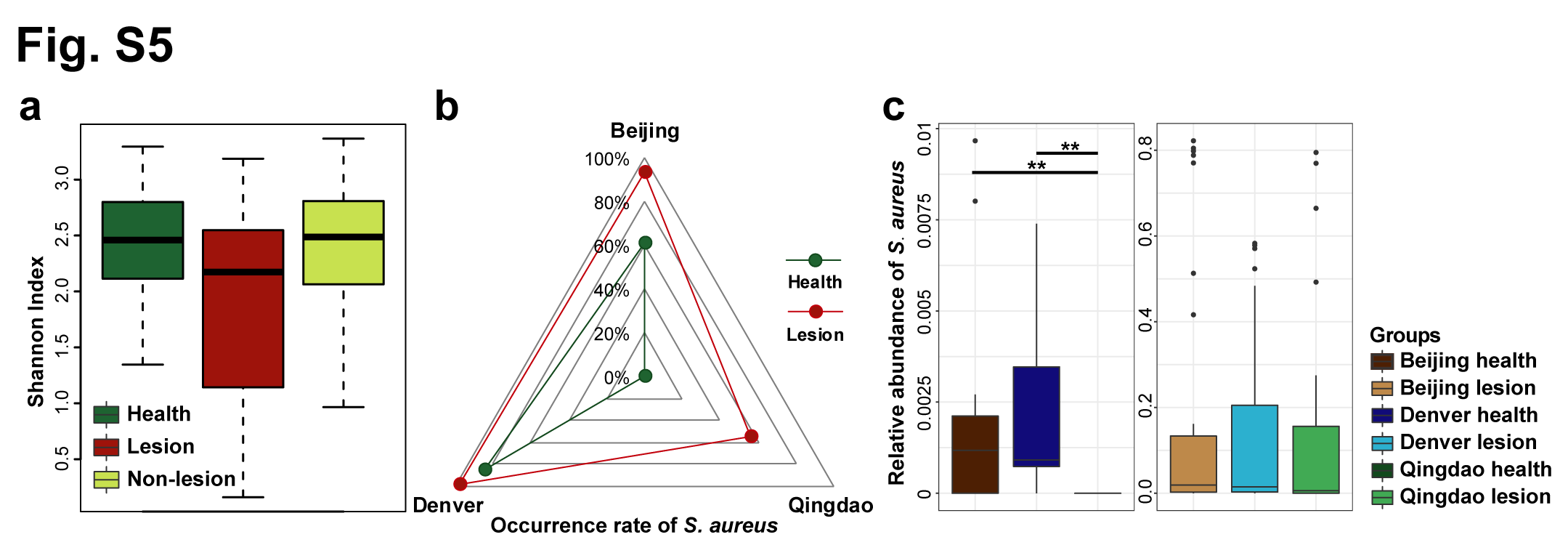

Supplement: FIG S5 [file mSystems.00293-19-sf005.tif]

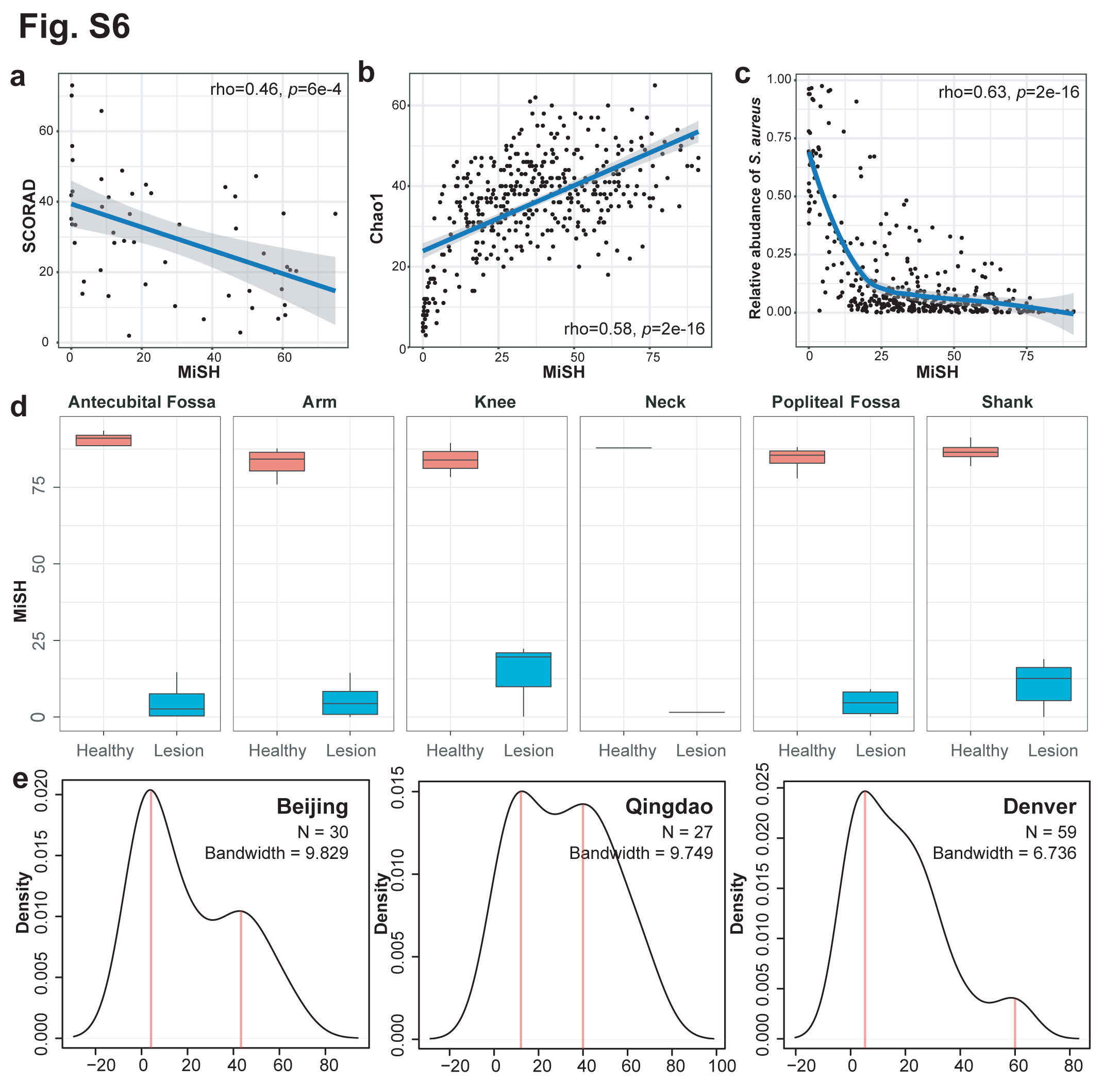

Supplement: FIG S6 [file mSystems.00293-19-sf006.tif]

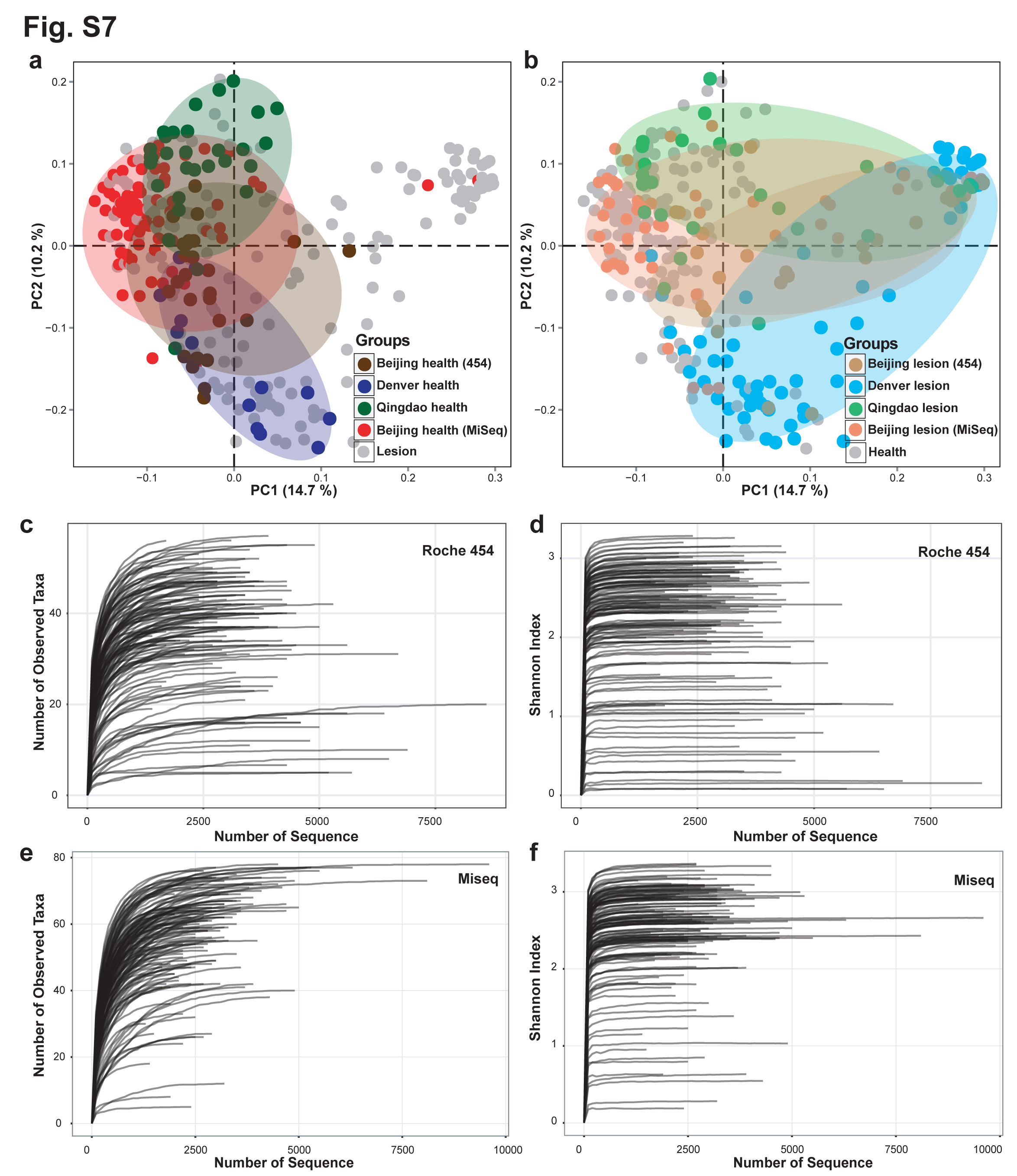

Supplement: FIG S7 [file mSystems.00293-19-sf007.tif]
