# Supplementary material for: A Microbiome-Based Index for Assessing Skin Health and Treatment Effects for Atopic Dermatitis in Children
Source: mSystems. 2019 Aug 20;4(4):e00293-19. doi: 10.1128/mSystems.00293-19 (PMC6702293; doi:10.1128/mSystems.00293-19)
Supplement: TABLE S2 [file mSystems.00293-19-st002.docx]

Supplementary Table2 for

**A Microbiome-based Index for Assessing Skin Health and Treatment effect for Atopic Dermatitis in Children**

Table S2. Differentially distributed genera among the three cities of Beijing, Qingdao and Denver in healthy and AD-active children.

**Table S2. Differentially distributed genera among the three cities of Beijing, Qingdao and Denver in healthy and AD-active children.**

| **Health** | **Average abundance** | | | **Statistical test** | |
| --- | --- | --- | --- | --- | --- |
|  | **Beijing** | **Denver** | **Qingdao** | ***p*-value** | **FDR** |
| *Corynebacterium.* | 0.0728 | 0.0207 | 0.0187 | 0.000 | 0.000 |
| *Staphylococcus.* | 0.0828 | 0.0416 | 0.0042 | 0.000 | 0.000 |
| *Streptococcus.* | 0.0797 | 0.2664 | 0.0377 | 0.000 | 0.000 |
| *Propionibacterium.* | 0.0525 | 0.1439 | 0.0186 | 0.000 | 0.000 |
| *Granulicatella.* | 0.0067 | 0.0236 | 0.0026 | 0.000 | 0.000 |
| *Kocuria.* | 0.0459 | 0.0096 | 0.0090 | 0.000 | 0.000 |
| *Actinomyces.* | 0.0143 | 0.0162 | 0.0040 | 0.000 | 0.000 |
| *Streptophyta_Group.* | 0.0169 | 0.0177 | 0.3202 | 0.000 | 0.000 |
| *Micrococcus.* | 0.0662 | 0.0075 | 0.0149 | 0.000 | 0.000 |
| *Lactobacillus.* | 0.0027 | 0.0023 | 0.0000 | 0.000 | 0.000 |
| *Paracoccus.* | 0.0421 | 0.0005 | 0.0142 | 0.000 | 0.000 |
| *Micrococcaceae_Group.* | 0.0093 | 0.0001 | 0.0004 | 0.000 | 0.000 |
| *Nocardioides.* | 0.0144 | 0.0096 | 0.0010 | 0.000 | 0.001 |
| *Ochrobactrum.* | 0.0004 | 0.0001 | 0.0064 | 0.000 | 0.000 |
| *Deinococcus.* | 0.0522 | 0.0009 | 0.0186 | 0.000 | 0.000 |
| *Paenibacillus.* | 0.0049 | 0.0007 | 0.0006 | 0.000 | 0.000 |
| *Bacillus.* | 0.0016 | 0.0014 | 0.0003 | 0.000 | 0.000 |
| *Veillonella.* | 0.0067 | 0.0161 | 0.0008 | 0.000 | 0.000 |
| *Caloramator.* | 0.0008 | 0.0059 | 0.0001 | 0.000 | 0.000 |
| *Gemella.* | 0.0131 | 0.0231 | 0.0021 | 0.000 | 0.000 |
| *Anaerococcus.* | 0.0047 | 0.0023 | 0.0006 | 0.000 | 0.000 |
| *Arthrobacter.* | 0.0038 | 0.0031 | 0.0004 | 0.000 | 0.000 |
| *Janibacter.* | 0.0302 | 0.0002 | 0.0117 | 0.002 | 0.004 |
| *Prevotella.* | 0.0009 | 0.0103 | 0.0006 | 0.000 | 0.000 |
| *Lactococcus.* | 0.0003 | 0.0150 | 0.0003 | 0.000 | 0.000 |
| *Citrullus.* | 0.0001 | 0.0000 | 0.0225 | 0.000 | 0.000 |
| *Stenotrophomonas.* | 0.0019 | 0.0018 | 0.0021 | 0.002 | 0.004 |
| *Agrobacterium.* | 0.0005 | 0.0013 | 0.0025 | 0.000 | 0.000 |
| *Haemophilus.* | 0.0001 | 0.0056 | 0.0071 | 0.000 | 0.000 |
| *Comamonas.* | 0.0018 | 0.0000 | 0.0054 | 0.000 | 0.000 |
| *Wautersiella.* | 0.0008 | 0.0000 | 0.0091 | 0.000 | 0.000 |
| *Acinetobacter.* | 0.0002 | 0.0081 | 0.1606 | 0.000 | 0.000 |
| *Enterobacter.* | 0.0001 | 0.0005 | 0.0251 | 0.000 | 0.000 |
| *Moraxella.* | 0.0000 | 0.0061 | 0.0350 | 0.000 | 0.000 |
| *Pseudomonas.* | 0.0000 | 0.0047 | 0.0172 | 0.000 | 0.000 |
| *Enhydrobacter.* | 0.0000 | 0.0060 | 0.0235 | 0.000 | 0.000 |
| *Carica.* | 0.0000 | 0.0000 | 0.0114 | 0.000 | 0.000 |
| *Alloiococcus.* | 0.0000 | 0.0022 | 0.0036 | 0.000 | 0.000 |
|  |  |  |  |  |  |
| **Lesion** | **Average abundance** | | | **Statistical test** | |
|  | **Beijing** | **Denver** | **Qingdao** | ***p*-value** | **FDR** |
| *Kocuria.* | 0.0194 | 0.0044 | 0.0192 | 0.000 | 0.000 |
| *Leptotrichia.* | 0.0008 | 0.0036 | 0.0019 | 0.000 | 0.000 |
| *Moraxella.* | 0.0000 | 0.0032 | 0.0046 | 0.000 | 0.000 |
| *Bergeyella.* | 0.0025 | 0.0012 | 0.0013 | 0.003 | 0.005 |
| *Prevotella.* | 0.0006 | 0.0136 | 0.0014 | 0.000 | 0.000 |
| *Streptococcus.* | 0.0627 | 0.2055 | 0.0300 | 0.000 | 0.000 |
| *Janibacter.* | 0.0071 | 0.0013 | 0.0037 | 0.001 | 0.001 |
| *Acinetobacter.* | 0.0000 | 0.0058 | 0.2213 | 0.000 | 0.000 |
| *Streptophyta_Group.* | 0.0062 | 0.0108 | 0.0345 | 0.000 | 0.000 |
| *Paracoccus.* | 0.0205 | 0.0009 | 0.0071 | 0.000 | 0.000 |
| *Micrococcus.* | 0.0386 | 0.0073 | 0.0341 | 0.000 | 0.000 |
| *Bacillus.* | 0.0155 | 0.0117 | 0.0045 | 0.004 | 0.006 |
| *Pseudomonas.* | 0.0000 | 0.0023 | 0.0085 | 0.000 | 0.000 |
| *Haemophilus.* | 0.0000 | 0.0049 | 0.0013 | 0.000 | 0.000 |
| *Enterobacter.* | 0.0000 | 0.0015 | 0.0323 | 0.000 | 0.000 |
| *Acaryochloris.* | 0.0056 | 0.1092 | 0.0003 | 0.000 | 0.000 |
| *Agrobacterium.* | 0.0000 | 0.0003 | 0.0039 | 0.000 | 0.000 |
| *Capnocytophaga.* | 0.0005 | 0.0025 | 0.0005 | 0.000 | 0.000 |
| *Ochrobactrum.* | 0.0000 | 0.0001 | 0.0137 | 0.000 | 0.000 |
| *Porphyromonas.* | 0.0012 | 0.0155 | 0.0013 | 0.000 | 0.000 |
| *Brevundimonas.* | 0.0040 | 0.0022 | 0.0127 | 0.000 | 0.000 |
| *Comamonas.* | 0.0001 | 0.0000 | 0.0097 | 0.000 | 0.000 |
| *Micrococcaceae_Group.* | 0.0092 | 0.0000 | 0.0004 | 0.000 | 0.000 |
| *Enhydrobacter.* | 0.0000 | 0.0029 | 0.0137 | 0.000 | 0.000 |
| *Paenibacillus.* | 0.0884 | 0.0061 | 0.0296 | 0.000 | 0.000 |
| *Neisseria.* | 0.0031 | 0.0232 | 0.0108 | 0.000 | 0.000 |
| *Caloramator.* | 0.0014 | 0.0029 | 0.0005 | 0.006 | 0.009 |
| *Granulicatella.* | 0.0046 | 0.0209 | 0.0014 | 0.000 | 0.000 |
| *Gemella.* | 0.0123 | 0.0199 | 0.0073 | 0.000 | 0.001 |
| *Stenotrophomonas.* | 0.0004 | 0.0007 | 0.0055 | 0.000 | 0.000 |
| *Brachybacterium.* | 0.0033 | 0.0012 | 0.0079 | 0.000 | 0.000 |
| *Fusobacterium.* | 0.0012 | 0.0041 | 0.0005 | 0.000 | 0.000 |
| *Prevotella.* | 0.0013 | 0.0040 | 0.0005 | 0.000 | 0.000 |
| *Actinomyces.* | 0.0033 | 0.0133 | 0.0020 | 0.000 | 0.000 |
| *Anaerococcus.* | 0.0005 | 0.0056 | 0.0014 | 0.006 | 0.009 |
| *Deinococcus.* | 0.0280 | 0.0006 | 0.0052 | 0.000 | 0.000 |
| *Peptoniphilus.* | 0.0003 | 0.0038 | 0.0004 | 0.002 | 0.003 |
| *Abiotrophia.* | 0.0001 | 0.0028 | 0.0001 | 0.000 | 0.000 |
| *TM7.* | 0.0032 | 0.0037 | 0.0003 | 0.001 | 0.001 |
| *Veillonella.* | 0.0035 | 0.0128 | 0.0011 | 0.000 | 0.000 |
| *Lactococcus.* | 0.0001 | 0.0043 | 0.0000 | 0.000 | 0.000 |
