# Supplementary material for: A Microbiome-Based Index for Assessing Skin Health and Treatment Effects for Atopic Dermatitis in Children
Source: mSystems. 2019 Aug 20;4(4):e00293-19. doi: 10.1128/mSystems.00293-19 (PMC6702293; doi:10.1128/mSystems.00293-19)
Supplement: TABLE S3 [file mSystems.00293-19-st003.docx]

Supplementary Table3 for

**A Microbiome-based Index for Assessing Skin Health and Treatment effect for Atopic Dermatitis in Children**

Table S3. Comparison of experimental protocols for skin microbiome sequencing among the three cities.

**Table S3. Comparison of experimental protocols for skin microbiome sequencing among the three cities.**

|  | **Beijing & Qingdao** | **Denver** |
| --- | --- | --- |
| **Swabs^a^** | Sterile flocked Swab | BD BBL Culture Swab |
| **Buffer^b^** | 0.15 M NaCl and 0.1% Tween 20 | ATL buffer (Qiagen, Inc., Valencia, CA) |
| **DNA extraction kit^c^** | Qiagen tissue and blood DNA isolation kit | Qiagen QIAamp DNA micro kit |
| **Primers** | 27F/534R | 27F/534R |
| **PCR amplification protocol^d^** | HMP # 07-001 | HMP # 07-001 |
| **Sequencing platform** | Illumina MiSeq (Paired-end reads) & Roche 454 | Illumina MiSeq (Paired-end reads) |

^a^: Both are DNA-free.

^b^: This buffer is for humidifying body sites before sampling.

^c^: Both kits provide fast and easy silica-based DNA purification for fresh or frozen blood, tissue and dried blood spots (https://www.qiagen.com/ca/shop/sample-techno-

-logies/dna/genomic-dna/).

^d^: https://www.hmpdacc.org/hmp/doc/HMP_MOP_Version12_0_072910.pdf
